# An initial investigation of accuracy required for the identification of small molecules in complex samples using quantum chemical calculated NMR chemical shifts

**DOI:** 10.1186/s13321-022-00587-7

**Published:** 2022-09-22

**Authors:** Yasemin Yesiltepe, Niranjan Govind, Thomas O. Metz, Ryan S. Renslow

**Affiliations:** 1 The Gene and Linda Voiland School of Chemical Engineering and Bioengineering, Washington State University, Pullman, WA USA; 2 Earth and Biological Sciences Directorate, Pacific Northwest National Laboratory, Richland, WA USA

**Keywords:** Metabolomics, Small molecules, NMR, DFT, Quantum chemistry

## Abstract

The majority of primary and secondary metabolites in nature have yet to be identified, representing a major challenge for metabolomics studies that currently require reference libraries from analyses of authentic compounds. Using currently available analytical methods, complete chemical characterization of metabolomes is infeasible for both technical and economic reasons. For example, unambiguous identification of metabolites is limited by the availability of authentic chemical standards, which, for the majority of molecules, do not exist. Computationally predicted or calculated data are a viable solution to expand the currently limited metabolite reference libraries, if such methods are shown to be sufficiently accurate. For example, determining nuclear magnetic resonance (NMR) spectroscopy spectra in silico has shown promise in the identification and delineation of metabolite structures. Many researchers have been taking advantage of density functional theory (DFT), a computationally inexpensive yet reputable method for the prediction of carbon and proton NMR spectra of metabolites. However, such methods are expected to have some error in predicted ^13^C and ^1^H NMR spectra with respect to experimentally measured values. This leads us to the question–what accuracy is required in predicted ^13^C and ^1^H NMR chemical shifts for confident metabolite identification? Using the set of 11,716 small molecules found in the Human Metabolome Database (HMDB), we simulated both experimental and theoretical NMR chemical shift databases. We investigated the level of accuracy required for identification of metabolites in simulated pure and impure samples by matching predicted chemical shifts to experimental data. We found 90% or more of molecules in simulated pure samples can be successfully identified when errors of ^1^H and ^13^C chemical shifts in water are below 0.6 and 7.1 ppm, respectively, and below 0.5 and 4.6 ppm in chloroform solvation, respectively. In simulated complex mixtures, as the complexity of the mixture increased, greater accuracy of the calculated chemical shifts was required, as expected. However, if the number of molecules in the mixture is known, e.g., when NMR is combined with MS and sample complexity is low, the likelihood of confident molecular identification increased by 90%.

**Supplementary Information:**

The online version contains supplementary material available at 10.1186/s13321-022-00587-7.

## Introduction

Metabolomics and exposomics involve the large-scale study of small molecules found in biological and environmental samples, including endogenous and exogenous chemicals, and their molecular breakdown products [1–3]. For human studies, understanding the active metabolic pathways and fate of exogenous chemicals is a major focus area for improving health through precision medicine, as well as an important tool for researching and understanding the state of environmental and agricultural conditions [4–7]. Biological and environmental samples typically comprise numerous molecules and are often in a complex matrix. It is not practical to develop or apply sample preparation methods for isolation of individual constituents (whether because of concentration limits, separation difficulty, or project cost limitations). The ability to comprehensively characterize such complex samples would result in significant advances in multiple scientific fields and enable currently irresolvable solutions for the understanding of metabolic pathways and biological systems such as active phenotypes/functions, industrial reactions such as they relate to (bio)fuels and high-value (bio)products, environmental processes, human actions in society, and even earth systems and climate.

Using nuclear magnetic resonance (NMR) [8–10], mass spectrometry (MS) [11–13], and other tools [14–16], a wide range of molecules have been identified and extensively documented in the literature [17–21]. Hundreds of thousands of metabolites are now known and their MS/MS or NMR data are electronically available on public and commercial chemical databases [22, 23] such as PubChem [24], Royal Society of Chemistry ChemSpider [25], ChEMBL by European Molecular Biology Laboratory [26, 27], Chemical Entities of Biological Interest (ChEBI) [28, 29], DrugBank [30, 31], Biological Magnetic Resonance Bank (BMRB) [32] and Human Metabolome Database (HMDB) [33], GDB13 [34], The Small Molecule Pathway Database (SMPDB) [35, 36], Distributed Structure-Searchable Toxicity (DSSTox) Database [37], E. coli Metabolome Database (ECMDB) [38, 39], EcoCyc E. coli Database [40], Food Component Database (FooDB) [41], LIPID MAPS In-Silico Structure Database (LMISSD) [42], MetaCyc Metabolic Pathway Database [43], MolMall [44], Super Natural II [45], The Toxin and Toxin Target Database (T3DB) [46, 47], ToxCast [48], The Universal Natural Products Database (UNPD) [49], ZINC [50]. However, the vast majority of molecules that are found in complex biological and environmental samples are not represented in current identification libraries (across multiple analytical platforms) [51, 52]. For example, the largest mass spectral library, the Wiley Registry and NIST Libraries contain more than 1 million mass spectra [53, 54]. HMDB (ver. 4.0) describes 114,260 metabolites, and of the molecules described in HMDB, only a small portion are available for purchase as authentic reference material [55–57]. ZINC 15, a database of ~ 1.8 B compounds, currently has 81,519 endogenous human metabolite structures, and of these, 9490 (12%) are immediately available for purchase [58, 59]. Furthermore, it is hypothesized that 10^60^ or more molecules are structurally feasible (for molecules < 1000 Da) [60–62], and much fewer than 1% are available in molecular identification reference libraries [63–65]. Thus, one cause of our current restricted size of small molecule identification libraries is due to the limited number of molecules available for purchase as authentic reference material. Even if all molecules were known and available for purchase, the time and cost to analyze these for building reference libraries would be prohibitive [66, 67]. The fields of metabolomics and exposomics, and small molecule identification generally, must overcome the significant, longstanding obstacle in the field: the absence of analytical methods for comprehensive and unambiguous identification of small molecules without reliance on reference data obtained from analysis of chemical standards [68–70].

For molecular properties that are consistently calculable with a known (preferably low) error, it is possible to create in silico reference libraries in order to reduce reliance on authentic chemical standards [70]. Several analytical methodologies, such as those based on chromatography coupled with MS[71–73] and NMR [74] have demonstrated feasibility for compound identification based on predicted properties. NMR’s ability to be non-destructive and easily quantifiable makes it a unique tool for identifying novel compounds and handling complex metabolite mixtures without the need of chemical separation [75]. For example, MS/MS spectra yield reasonable accuracy for predictions of molecular properties and can be coupled with machine learning methods [76] but limited to short lists of small molecules [77, 78]. Quantum chemical applications such as infrared spectra [79], molecular collisional cross sections (CCS) [80, 81] and NMR chemical shifts [82–85], are promising for the calculations of molecular attributes. For example, coupling calculated mass and CCS has contributed to successful chemical identification of cis/trans isomers [86, 87], as well as isomers in complex synthetic samples [88]. For studies specifically using NMR, quantum chemical simulations for the prediction of spectra have been a valuable tool for the community. In the last two decades, density functional theory (DFT), an exceptionally well-established approach for high-throughput chemical calculations with the advantage of high performance for less computational cost, has been widely applied to predict NMR chemical shifts [89–91] of molecules and conformers [92–94] in different custom solvent conditions [95–97]. Furthermore, structural elucidation is one of the most practical uses of NMR, and it is common to utilize NMR chemical shift calculations along with experimental shifts to identify compound mixtures [98–100] and to aid reassignment of structures or stereostructure assignment [101–103].

Currently, the use and acceptance of predicted NMR chemical shifts is limited due to an incomplete understanding of the required accuracy of such predictions for confident molecular identification. It has already been demonstrated that heuristic/empirical approaches for chemical shift predictions are generally of low accuracy compared to quantum chemical calculation-based methods (e.g. DFT) [83, 104, 105]. For DFT approaches, the factors that significantly affect the accuracy of predicted ^13^C and/or ^1^H NMR chemical shifts are the optimization level of molecular geometry [106–108], the use of different DFT theories [109–112], implicit and explicit solvation models [113–115], unique molecular properties of metabolites [116–121], etc. Agreement between predicted and experimental chemical shifts can be improved when (i) the basis set is enlarged [104, 122], (ii) the quality of the method is improved for geometry optimization [123, 124], (iii) a scaling procedure is employed [125, 126], (iv) conformational sampling is applied [127], and (v) solvation is taken into account appropriately [128, 129]. However, the question of what level of accuracy is required for calculated NMR chemical shifts when using these as reference spectra for molecular identification remains largely unexplored.

In this study, we investigate the accuracy and/or level of confidence in predicted NMR chemical shifts required to identify small molecules using reference libraries of varying size. Specifically, we present a detailed study on the role of accuracy in the prediction of ^13^C and ^1^H NMR spectra for confident metabolite identification in solution phase using a chloroform and water continuum model. We estimate the minimum and maximum error limits which hinder or enable ^13^C and ^1^H NMR chemical shift predictions to unambiguously identify molecular structures. In this study, we discuss two cases—simple and complex samples—using 11,716 small molecules taken from the HMDB [18]. We cover different chemical functional groups and explore the results to provide statistics for libraries of different sizes.

## Materials and methods

### Molecule sets

**Fig. 1:**
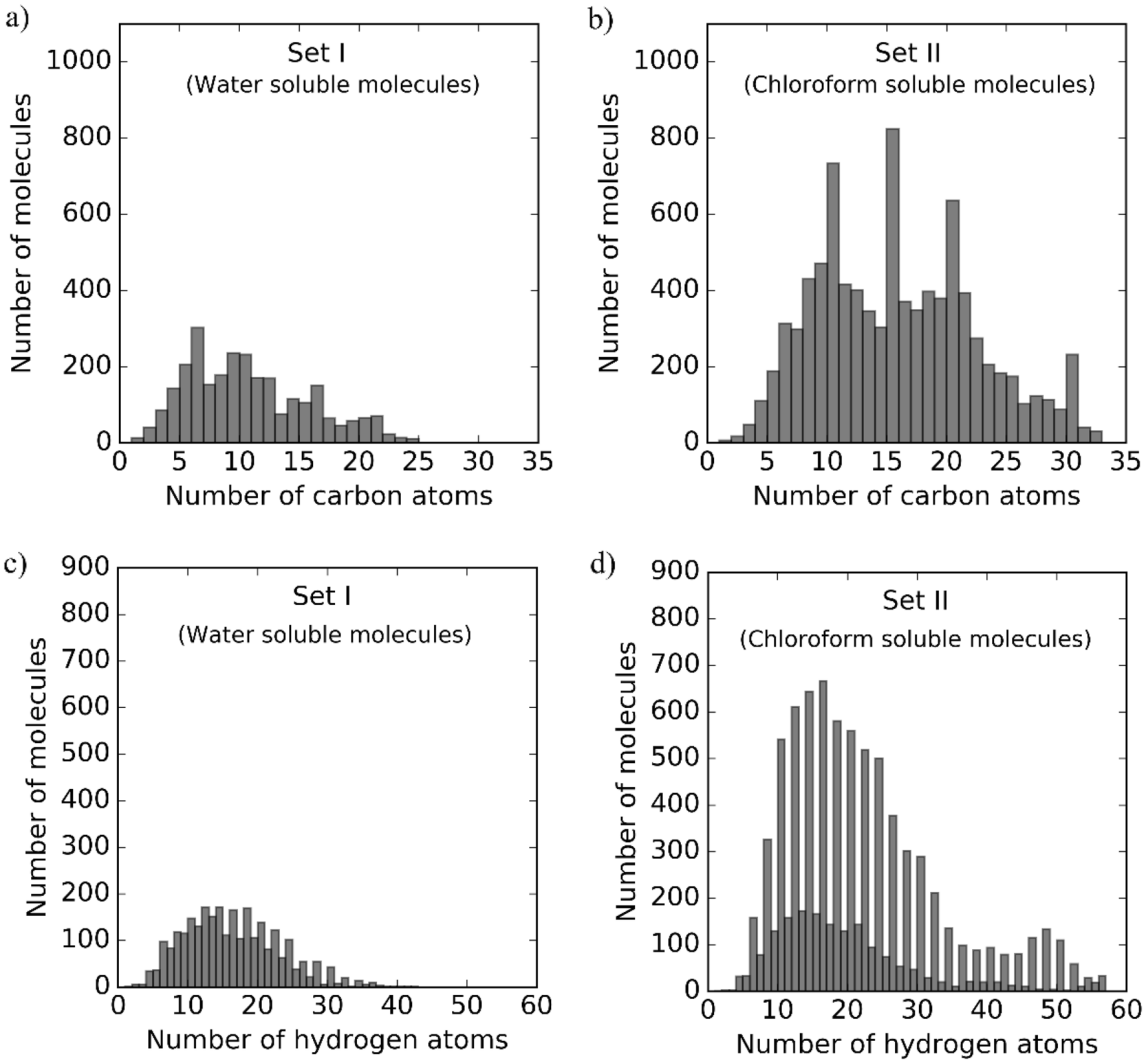
Histograms depicting the number of molecules in each set for a given number of carbon atoms in **a** Set I and **b** Set II, and hydrogen atoms in **c** Set I and **d** Set II

Two sets of molecules taken from HMDB 4.0 [56] and distinguished by their reported partition coefficients were simulated, one in water (Set I) and a second in chloroform (Set II) as the solvent. The included compounds were not in salt forms, consist only of C, H, O, N, P and S atoms, and are in the molecular weight range of 27 to 500 Da. Set I, the water solvated set, contains 2,723 molecules (29,489 carbon and 45,426 hydrogen nuclei in total across all molecules) and spans a wide range of structure-based chemical classes and chemical functionalities including organic acids, organonitrogen compounds, nucleosides, nucleotides, organoheterocyclic compounds, carboxylic acids, organooxygen compounds, and benzenoids as determined by the hierarchical chemical classification scheme, ClassyFire [130]. Set II, the chloroform solvated set, contains 8,990 molecules (138,535 carbon and 191,327 hydrogen nuclei in total across all molecules) and also spans a broad range of chemical functionalities including organic compounds, organic acids, lipids, benzenoids, and organoheterocyclic compounds. Figure 1 compares the number of molecules containing a given amount of carbons and hydrogens for Sets I and II. The molecules and their geometries in both Sets are provided in the Additional file 1.

**Fig. 2:**
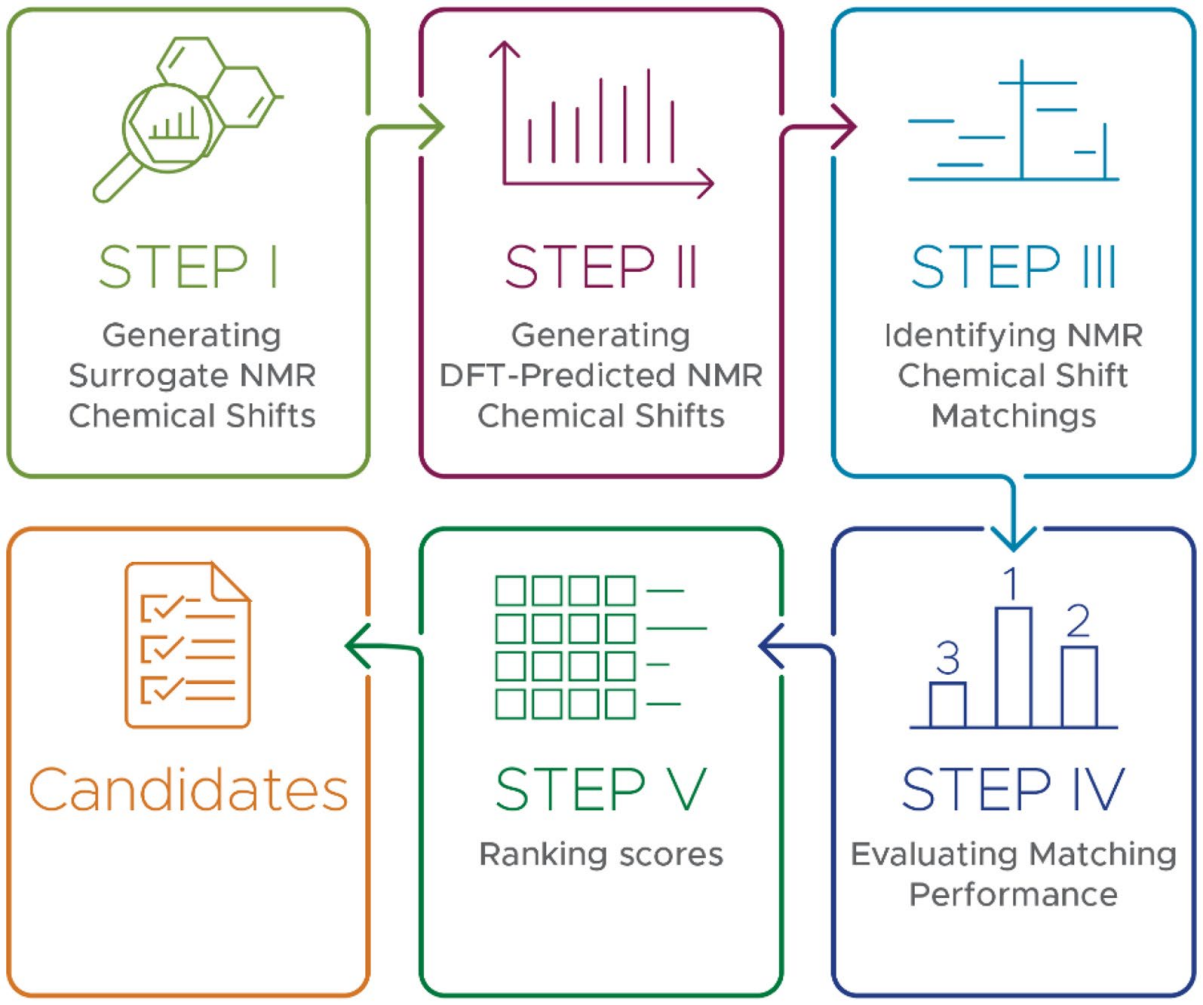
Flowchart for the identification of the compounds in a mixture

### Computational details

The NMR chemical shifts for all molecules in this study were calculated using the In Silico Chemical Library Engine (ISiCLE) [131] (see github.com/pnnl/isicle for the latest version of ISiCLE). ISiCLE is an automated pipeline for high-accuracy chemical property calculation, implemented using the Snakemake workflow management system [132]. This pipeline takes SMILES [133] (a line notation representation of molecule structure) as input, generates initial 3D molecular conformations, and subsequently optimizes this initial structure and calculates chemical properties through quantum chemistry via NWChem [134] (an open-source, high-performance computational chemistry software developed at PNNL). For this study, all molecules were initially optimized in solvent using the computationally inexpensive B3LYP [135, 136] with 3-21G basis set [137–139]. We chose this level of theory due to our available computational resources, particularly considering the treatments for the geometry optimization of over 11 k molecules. It is known that the 3-21G basis set for geometry optimization is not adequate to obtain high accuracy in NMR chemical shift calculations [127, 140, 141], but in this study it is only used to simulate NMR spectral data in order to obtain a reasonable representative distribution of (likely moderate accuracy) chemical shifts. Assessment of the best computational approaches to maximize accuracy of NMR chemical shift calculations is beyond the scope of this study. To test whether the NMR spectral data is statistically affected or not by using any other DFT method, the isotropic shielding values of 5 randomly chosen molecules in different shapes and sizes from Sets I and II were calculated using 3 different DFT methods. The shielding values were observed to be shifted in the same direction following the same pattern. Further details are given in Additional file 2. The inclusion of solvent is via the COnductor-like Screening MOdel (COSMO) [142] solvation modeling. NMR isotropic shieldings were calculated for all optimized molecules at the B3LYP/cc-pVDZ [139, 143] level of theory. Based on our previous assessment [131], this method provides reliable chemical shifts [112] and yields isotropic shieldings with a reasonably low computational cost [144]. The gauge-invariant atomic orbital (GIAO) approach [145] was used to compute ^13^C and ^1^H NMR chemical shifts. The computed chemical shifts are provided in Additional file (available upon author request).

### Algorithm

Various scoring approaches have been proposed for the analysis of chemical shifts and comparisons of DFT methods. The most common criteria in the literature quantifying the agreement between calculated and experimental data are mean absolute error (MAE) (Eq. 1), root mean square error (RMSE) (Eq. 2), corrected mean absolute error (CMAE) (Eq. 3), and correlation coefficients (e.g., the Pearson correlation coefficient).

1 $${\text{MAE}} = \frac{{\mathop \sum \nolimits_{{{\text{i}} = 1}}^{{\text{N}}} \left| {{\updelta }_{{{\text{exp}}}} - {\updelta }_{{{\text{calc}}}} } \right|}}{{\text{N}}}$$

2 $${\text{RMSE}} = \sqrt {\frac{{\mathop \sum \nolimits_{{{\text{i}} = 1}}^{{\text{N}}} \left( {{\updelta }_{{{\text{exp}}}} - {\updelta }_{{{\text{calc}}}} } \right)^{2} }}{{\text{N}}}}$$

3 $${\text{CMAE}} = \frac{{\mathop \sum \nolimits_{{{\text{i}} = 1}}^{{\text{N}}} \left| {{\updelta }_{{{\text{exp}}}} - ({\updelta }_{{{\text{calc}}}} - {\text{b}})/{\text{m}}} \right|}}{{\text{N}}}$$

where ${\updelta }_{\mathrm{exp}}$ is the experimental chemical shift, ${\updelta }_{\mathrm{calc}}$ is the calculated chemical shift, $\mathrm{N}$ is the number of nuclei, and $\mathrm{m}$ and $\mathrm{b}$ denote slope and intercept of the calculated shifts with respect to experimental shifts.

To identify the compounds in a mixture, our approach follows the steps in the flowchart presented in (Fig. 2). In Step I, NMR chemical shifts of all molecules are calculated as described above. Since we do not have experimental NMR data for the 11 thousand molecules in our two sets, in step II we create representative NMR data for comparisons: the calculated NMR spectra (generated in Step I) are considered as surrogate experimental shifts data and new lists of chemical shifts are created synthetically by adding Gaussian distributed noise. Although the error distributions of NMR chemical shifts were reported to also obey a student t-distribution in other studies [131, 146–149], we assume errors for both carbons and protons follow a Gaussian distribution [144, 150] with mean µ and standard deviation σ. Unless otherwise stated, the mean is assigned as 0, since the errors of scaled ^13^C and ^1^H NMR chemical shifts are equally likely to be positive or negative [144, 147]. In this study, σ is taken in the range of 0.5–50 ppm and 0.1–10 ppm for ^13^C and ^1^H chemical shifts with increment of 0.05 ppm and 0.01 ppm, respectively. Simply, we assume that our initial (non-noise-added) calculated chemical shifts (“surrogate experimental data”) represent the distribution, but not necessarily the accuracy of authentic experimental chemical shifts, and that the addition of zero-mean Gaussian noise to create synthetic data with a defined error allows us to explore how the accuracy of real calculated chemical shifts can affect identification rates. This approach is similar to that taken in other successful studies [151–153]

In Step III, each molecule taken from the computed data is searched back against the surrogate experimental data. First, the experimental chemical shifts of an unknown molecule are matched to the computed chemical shifts of every single molecule to find the best match, based on minimizing the distance between two sets of chemical shifts. To do this, we used the Munkres assignment algorithm [154–156], which gives the minimum distance score (i.e. error) of two sets, within a feasible computational time bounded by a polynomial expression [157]. The Munkres algorithm minimizes the total error or summation of squared differences between each assignment. It is based on the following principle:

Let S1 and S2 be two separate lists of chemical shifts consisting of N and M elements, respectively. Let us construct an M-by-N matrix

$$\left[ {\begin{array}{*{20}c} {\left( {{\text{s}}_{1} - {\text{b}}_{1} } \right)^{2} } & \cdots & {\left( {{\text{s}}_{{\text{M}}} - {\text{b}}_{1} } \right)^{2} } \\ \vdots & \ddots & \vdots \\ {\left( {{\text{s}}_{1} - {\text{b}}_{{\text{N}}} } \right)^{2} } & \cdots & {\left( {{\text{s}}_{{\text{M}}} - {\text{b}}_{{\text{N}}} } \right)^{2} } \\ \end{array} } \right]$$

where s_i_ is the mth element of S1, b_j_ is the nth element of S2 and M ≤ N. We have M elements to be assigned to N elements on a one-to-one basis where the assignments constitute an independent set of the M-by-N matrix. Then, the Munkres algorithm models an assignment problem, which returns the least-sum of elements of the matrix, choosing only one element from each row and column. In our case, this indicates the best possible matching, which will be used in the next step.

In Step IV, for each molecule, to determine which set of experimental data best matches to the computed one, the similarity of two sets of assigned chemical shifts is quantified by a distance score. There is no perfect score (i.e. zero error) between two sets (e.g., in practice, there is always some amount of error expected between experimental and computed shifts). A critical issue is finding a method to quantify the error such that it always yields the best match at the top when the list of scores are sorted from most to least likely. In addition to the most popular ways to express chemical shift errors (i.e. MAE and RMSE), we believe that an indication of how confident a matching set is can be expressed better in terms of RMSE and probability. Smith et al. performed a sophisticated systematic study for addressing the issue of the best parameter, and proposed DP4 [147], which is used when experimental NMR data is to be used to identify one molecule out of an arbitrarily large library of many possible structures. DP4 is based on conditional probability and/or Bayes’ theorem—the key factor increasing the certainty of results. While we found DP4 to give slightly better rankings for pure samples than RMSE, we also found it to be computationally much more intense than RMSE. We also believe DP4 is not convenient for ranking matches in impure/complex samples. Therefore, we use RMSE in this study. Further details are given in Additional file 1.

Note that the RMSE ranges differ for carbon and proton. For the cases when carbon and proton are used together for identifying molecules, each RMSE is calculated separately and their geometric means are taken to get a single score for the molecule. The geometric mean is used to normalize the RMSEs, so the error associated with carbon does not dominate that of the proton for cases where both nuclei are used together.

Finally, in Step V, all resulting scores are sorted in ascending order, yielding a list of molecules starting from the most likely to the least likely to be found in the mixture. The ranks and scores of each molecule are reported. In this study, a rank of 1 (top of list) is synonymous with positive molecule identification.

For this study, we considered the case when (1) proton chemical shifts are used alone for identification, (2) carbon chemical shifts are used alone, and finally, (3) when both nuclei are used together.

The automated workflow and all scripts, written in Python, are provided in Additional file 4.

## Results and discussion

Robust and comprehensive metabolite identification using calculated NMR chemical shifts requires assessments of the accuracies of the in silico approaches used and that must have validated error ranges. We investigated the level of accuracy required to identify small molecules in NMR libraries. We performed a comprehensive analysis on the extent of accuracy in the predicted ^13^C and ^1^H NMR chemical shifts using 11,716 small molecules taken from the HMDB. We analyzed the limits (upper and lower) of error for confident metabolite identification. in two solution phases: chloroform and water. We discussed the possible error ranges in predicted NMR chemical shifts allowing to achieve reasonably confident identification in 2 types of samples: (i) pure uniform sample, and (ii) complex sample. We performed our runs for 190 different error ranges (i.e. σ, Gaussian standard deviation) and repeated the experiments 16 times for each case. Unless otherwise stated, all analyses were performed for each molecule in the two sets. We report the average results for i) ^13^C chemical shifts alone, ii) ^1^H chemical shifts alone, and iii) ^13^C and ^1^H chemical shifts used together for identification. We report the average percentage of molecules successfully identified (i.e. rank is 1) for Set I (water soluble molecules) and Set II (chloroform soluble molecules).

### Case I: Pure sample

In this case, let us assume we have a spectrum from a single compound and an array of carbon (^13^C) and/or proton (^1^H) NMR chemical shifts. This case involves selecting only the molecules having the exact number of carbon and/or proton chemical shifts from the database to match the experimental spectrum. This narrows the list of candidate molecules.

**Fig. 3:**
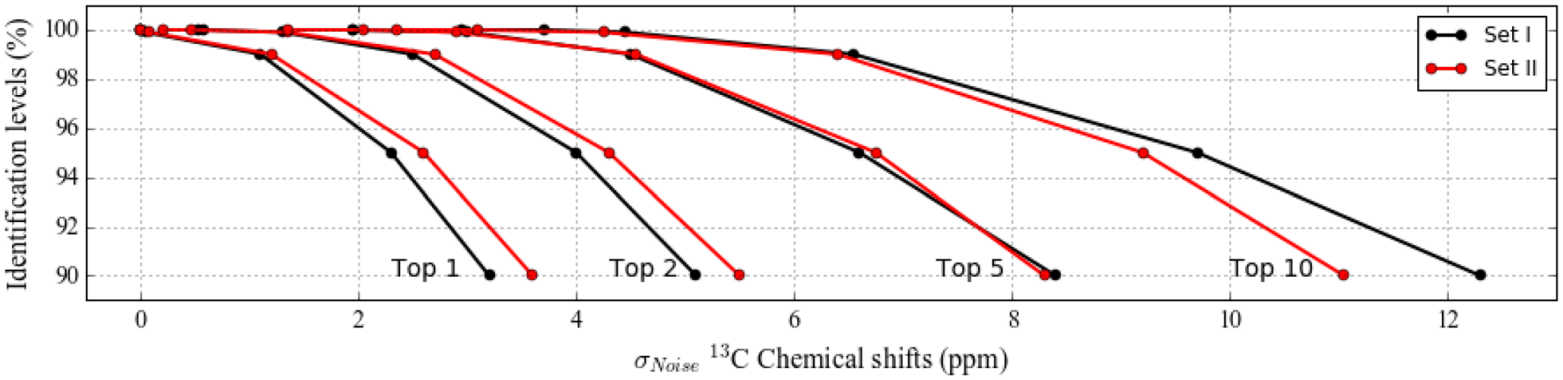
Averaged percentages of molecules being identified within the first 1, 2, 5, and 10 candidate molecules at different Gaussian standard deviation (σ) values (ppm) for Set I and Set II when ^13^C NMR chemical shifts are used alone

**Fig. 4:**
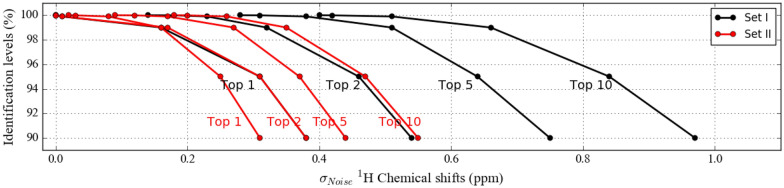
Averaged percent of molecules being identified within the first 1,2,5, and 10 candidate molecules at different Gaussian standard deviation (σ) values (ppm) for Set I and Set II when ^1^H NMR chemical shifts are used alone

Figures 3 and 4 show the identification results of 90% to 100% of the molecules of both sets in the top 10 hits (Top 10) for carbons and protons used independently. As an example, for identifying 90% of the molecules in the first hit (Top 1), ^13^C chemical shift errors should be below 3.2 and 3.6 ppm for Set I and Set II, respectively. Likewise, for ^1^H chemical shifts, when the MAE is at most 0.38 ppm for the both sets, there is a 90% chance that the correct identification will be made as the first hit. It is possible to correctly identify 99% of the molecules when the noise is at most 1.1–1.2 and 0.16–0.17 ppm for ^13^C and ^1^H chemical shifts, respectively. The molecule of interest has a chance to be among the first two candidate matches (Top 2) when ^13^C and ^1^H chemical shift errors are 0.53 ppm and 0.21 ppm, and 0.14 ppm and 0.02 ppm for Set I and Set II, respectively. However, for these sets of molecules, 100% of identification is not possible when ^13^C and ^1^H chemical shifts are used alone. The higher quality versions of Figs. 3 and 4, and the full list including 50–100% of identification is given in the Additional file (available upon author request).

**Fig. 5:**
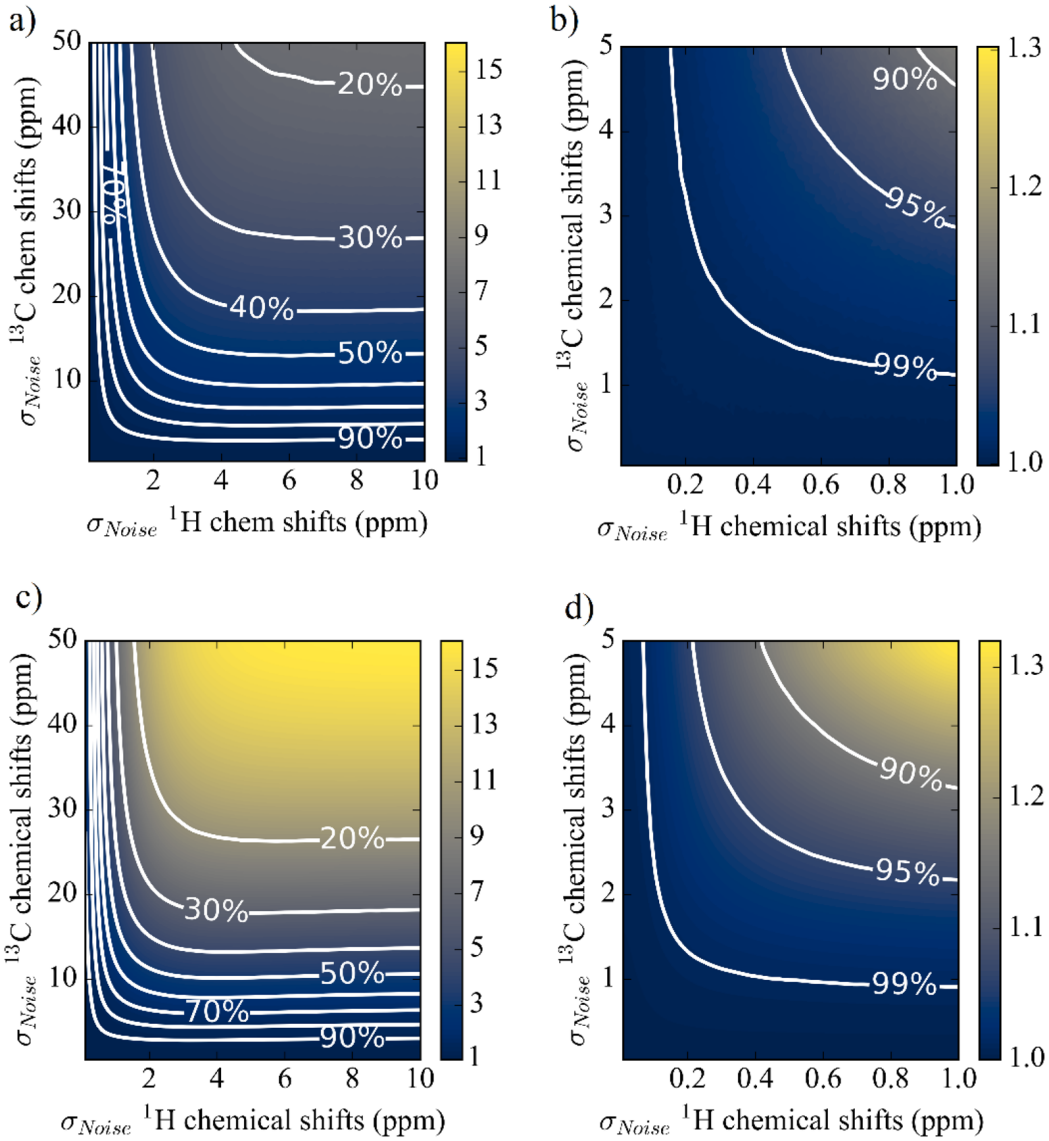
Mean of ranks with respect to the carbon and proton errors and contour lines for the different level of identification ratios when carbons and protons are used together for **a**, **b** Set I (water soluble molecules) and **c**, **d** Set II (chloroform soluble molecules). **b** and **d** are the zoomed versions of **a** and **c**, respectively. The color bars represent the rankings

Figure 5 shows where the molecules rank in identification lists for a comprehensive identification analysis for Set I and Set II, plotted against carbon and proton errors when ^13^C and ^1^H data are used together for identification. The plots show how the probability of a molecule being correctly identified changes with chemical shift errors. The contour lines represent different levels of identification with respect to carbon (y-axis) and proton (x-axis) errors. The color bars show the ranking distributions along the ranges of carbon (0–50 ppm) and proton errors (0–10 ppm). The contour lines are represented in a reciprocal relationship (Eq. 1) (see Additional file 3 for further information). Therefore, on each contour line, it is possible to have a list of combinations for a range of carbon and proton errors. For example, for 90% of identification, the carbon and proton errors (ppm) could be (3 and 10), (5 and 0.92), or (6 and 0.7), respectively, out of many combinations. This reciprocal relationship also gives a trade-off between the carbon and proton errors such that it is possible to skip expensive ^13^C chemical shifts over highly accurate ^1^H chemical shifts, and vice versa.

4 $${\text{MAE}}\left( {13{\text{C}}} \right) = {\text{a}}/\left( {{\text{MAE}}\left( {1{\text{H}}} \right) - {\text{b}}} \right) + {\text{c}}$$

Each contour line has an optimum point which represents a trade-off point (reported in Table 1). At these points on the curves, the cumulative errors of carbon and proton are minimum (note that ranges for carbon and proton errors are normalized). A fascinating but not unexpected observation here is that the chances of molecules being successfully identified are doubled when ^13^C and ^1^H chemical shifts are used together. Thus, compared to the previous case when ^13^C and ^1^H chemical shifts are used independently, using more information increases the chance of successful identification. The full list of trade-off points including 50–99% is reported in the Additional file (available upon author request).

It is observed the ranks range from 1 to 7 for Set I and 1 to 16 for Set II. The difference in ranges source from different sized molecule sets and differences in standard deviation and variance of ^13^C and ^1^H chemical shifts. The standard deviations of ranks are shown in the Additional file 2.

**Table 1:** Optimum trade-off MAEs at different Gaussian standard deviation (σ) values (ppm) for Set I and Set II when 13C and 1H NMR chemical shifts are used together for identification

Percentile (%)	Set I (Water soluble molecules) σ (ppm)		Set II (Chloroform soluble molecules) σ (ppm)	
	^13^C	^1^H	^13^C	^1^H
99	2.02	0.30	1.64	0.43
95	4.21	0.57	4.44	0.53
90	6.16	0.72	5.82	0.70

### Case II: Impure sample

A continuing grand challenge for NMR-based metabolomics is dealing with the spectral complexity in analysis of mixtures. An NMR spectra can have a combination of thousands of distinct resonances belonging either to the main compound or to impurities. Here, we used an approach very similar to a quantitative metabolomics approach in which identification and quantification are based on the underlying assumption that any given sample spectrum is the sum of individual spectra of pure metabolites found in the mixture. The spectrum of interest is compared to a library of pure compound spectra by properly matching and fitting the reference peaks. The reference libraries need to be prepared from NMR spectra of pure metabolites at a precisely known and controlled pH and temperature. Especially, peaks of water or some endogenous metabolite are pH, temperature and salt-sensitive, which frequently leads to errors. In this study, we disregarded the effects of pH and temperatures, and distortions, artifacts and noise in signals. We performed our analysis based on the assumption that the spectrum of every single compound in the mixture is a sub-spectrum stored in the reference database.

Let us assume we have an impure sample consisting of unknown number of compounds and carbon and/or proton NMR chemical shift data for the sample. In contrast to Case I, here we consider an n-tuple of molecules to be the list of candidates in the sample consisting of n number of molecules. Unlike Case I, the sequence of chemical shifts to be matched in the reference library do not necessarily have the same size of candidates; instead any molecule having equal or less ^13^C and ^1^H NMR chemical shifts in the reference library has a chance to be a candidate. For instance, if we have a sample of 2 molecules with c_1_ and c_2_ number of carbons and h_1_ and h_2_ number of protons, respectively, only the pairs having a sum of c_1_ + c_2_ carbons and h_1_ + h_2_ protons are the candidates and the chemical shifts of an atom can only belong to one of two candidates.

**Fig. 6:**
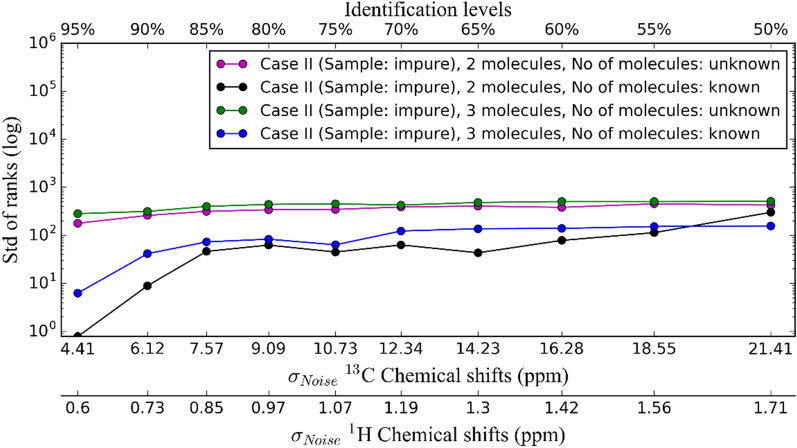
Average ranks of Case I and Case II with known/unknown number of molecules in samples at optimum points for Set I (water soluble molecules)

Compared to Case I, not only does the list of candidate molecules expand but matching two sets of data of different size is also not straightforward, making it even more challenging. Because of this, we did not examine this case for different Gaussian noise levels in detail as we did in Case I. We performed our runs for mixtures of 2 and 3 compounds. We report the results of this case only for a specific set of Gaussian noises (the optimum trade-off MAEs of ^13^C and ^1^H NMR chemical shifts reported for Set I in Table 1). Unless otherwise specified, we refer the mixtures of 2 and 3 compounds as pairs and triplets, respectively. In Fig. 6, the averaged ranks are shown for molecule pairs and triplets for all the optimum MAEs. Compared to the case of pure samples (Case I), the probability of identification decreases from 95 to 0% (pairs) and 6% (triplets) when the ^13^C and ^1^H NMR chemical shift errors are 4.41 ppm and 0.6 ppm, respectively. So, the identification chance is quite low (green and purple lines in Fig. 6) even when the ^13^C and ^1^H NMR chemical shift errors are low. We then investigated what happens if the number of compounds in the sample is known. At first this seems counter intuitive, but the probability of identification is increased to 84% (from 0%—pairs) and 68% (from 6%—triplets) when the ^13^C and ^1^H NMR chemical shift errors are 4.41 ppm and 0.6 ppm, respectively. The average identification chances increase by 83% and 91% (blue and black lines in Fig. 6). Determining the number of compounds in a sample may be possible using additional orthogonal data. For example, multidimensional NMR experiments or MS may aid in determining the number of high concentration molecular candidates in a sample. Integrating NMR and MS can provide improved identification and quantification of a larger number of metabolites, as in Case II. [158, 159]. This is still, however, less than Case I by 84% and 93% for pairs and triplets, respectively (red line in Fig. 6). Standard deviations of ranks and computational times of runs are given in the Additional file 1.

Case II was performed only for the smaller molecule set, Set I (water soluble molecules), and not for the larger set, Set II (chloroform soluble molecules), due to the high computational time demands.

NMR spectroscopy is one of the main methods used for identifying the structure of metabolites. Besides the usual parameters (i.e. ^13^C and ^1^H NMR chemical shifts), other major NMR parameters (i.e. spin–spin coupling constants and ^15^ N, ^17^O, and other nuclei chemical shifts) can alternatively be used for structure identification. We believe the use of any other property will significantly improve molecular identification. In this initial study, we did not test the effect of using additional information that can be collected using NMR (e.g., J-couplings and peak shape). However, most currently available databases provide only ^13^C and ^1^H NMR chemical shifts, and J-couplings, multi-dimensional spectra, etc. are missing for many molecules. There is rapid progress in the use of 2D NMR models (i.e. COSY, HSQC, and HMBC) which aids interpretation of spectrum and leads to less ambiguity in the spectral assignments and allows more reliable identification. 2D NMR techniques are proven to overcome the problem of insufficient spectral resolution and spectral redundancy. 2D NMR experiments provide additional information (i.e. couplings between magnetic nuclei) and solve the problem of overlapping peaks. Thus, it allows identification of metabolites that otherwise remain undetected. Multi-dimensional spectra prediction can be obtained using spin dynamics simulation libraries (i.e. SPINACH [160]) coupled with DFT calculations. We are currently assessing the present limits of such automated workflows for accelerating confident, accurate, and fast metabolite identification.

## Conclusion

Global comprehensive compound identification in complex samples will revolutionize understanding of the role of important compounds in chemical, environmental and biological studies. A major limitation is that the vast majority of metabolites are not available in current identification libraries, nor available for purchase as authentic reference material. It is not economically and practically feasible to identify hundreds of thousands of metabolites in laboratories to establish small molecule reference libraries. To address this, in silico small molecule libraries are currently the only reasonable solution to move toward comprehensive identification of all molecules in complex samples.

We performed an extensive statistical analysis on the effect of ^13^C and ^1^H NMR chemical shift calculation errors, in water and chloroform solvents, on the ability to make correct identification from in silico libraries. For pure samples, the required accuracy levels are feasible, promising the establishment of large scale metabolomic NMR in silico libraries. 90% or more of these molecules in a pure sample can be successfully identified when errors of ^13^C and ^1^H NMR chemical shifts are below 6 ppm and 0.5 ppm, respectively. This shows great potential of future use and reliability of predicted NMR chemical shifts in molecule identification for pure samples.

Compared to pure sample identification, it may require complementary information for complex samples in order to correctly identify constituent compounds. The water-soluble molecules in a complex sample have a chance of 68% and 84% (it is 95% for pure samples) to be identified for pairs and triplets, respectively when errors of ^13^C and ^1^H NMR chemical shifts are below 4.41 ppm and 0.6 ppm. The possibility of identification increases by 90% when the number of molecules are known beforehand, corroborating other findings that significant potential for parallel MS analysis [161]. This increased confidence in our results indicates the value of adding multiple molecular or chemical properties and using additional measured or accurately predicted information for comprehensive identification of metabolites.

This study provides valuable insight into the practicality and applicability of potential in silico small molecule NMR databases. The rapid innovations in metabolite identifications through the recent advances in computation and data integration in both NMR and MS/NMR analytical and computational methods will aid the full metabolome composition assignment in complex sample identification.

## Supplementary Information

**Additional file 1**. Set I - Water Soluble Molecules.

**Additional file 2**. Set II - Chloroform Soluble Molecules.

**Additional file 3**. Python Scripts.

**Additional file 4**. Supplementary Information Document.

## Data Availability

Molecule MOL files and DFT output files are included in the Additional files, along with Python processing code. Any other data is freely available upon request. The Additional files are available from the authors, upon request.
