# Supplementary material for: An initial investigation of accuracy required for the identification of small molecules in complex samples using quantum chemical calculated NMR chemical shifts
Source: J Cheminform. 2022 Sep 22;14:64. doi: 10.1186/s13321-022-00587-7 (PMC9499888; doi:10.1186/s13321-022-00587-7)
Supplement: Supplementary file 4 — Additional file 4. Supplementary Information Document. [file 13321_2022_587_MOESM4_ESM.docx]

**Supplementary Data**

An initial investigation of accuracy required for the identification of small molecules in complex samples using quantum chemical calculated NMR chemical shifts

**Authors:**

Yasemin Yesiltepe^1,2^, Niranjan Govind^2^, Thomas O. Metz^1^, Ryan S. Renslow^1,2^*

1 The Gene and Linda Voiland School of Chemical Engineering and Bioengineering, Washington State University, Pullman, WA, USA.

2 Earth and Biological Sciences Division, Pacific Northwest National Laboratory, Richland, WA, USA

*Corresponding author: Ryan Renslow ([ryan.renslow@pnnl.gov](mailto:ryan.renslow@pnnl.gov))

# **S1.** **Results of Case I**

Table 1. Averaged percent of molecules being identified with respect to Gaussian standard deviation values.

| **Percentile (%)** | **σ (ppm) - ^13^C** | | **σ (ppm) - ^1^H** | |
| --- | --- | --- | --- | --- |
|  | **Set I** | **Set II** | **Set I** | **Set II** |
| 100 | 0 | 0 | 0 | 0 |
| 99.99 | - | - | - | - |
| 99.9 | 0.04 | 0.08 | 0.01 | 0.01 |
| 99 | 1.1 | 1.2 | 0.16 | 0.17 |
| 95 | 2.3 | 2.6 | 0.31 | 0.31 |
| 90 | 3.2 | 3.6 | 0.38 | 0.38 |
| 85 | 3.9 | 4.4 | 0.44 | 0.45 |
| 80 | 4.6 | 5.1 | 0.49 | 0.5 |
| 75 | 5.4 | 5.8 | 0.54 | 0.55 |
| 70 | 6.1 | 6.5 | 0.59 | 0.59 |
| 65 | 7 | 7.1 | 0.64 | 0.64 |
| 60 | 7.9 | 7.9 | 0.69 | 0.69 |
| 55 | 8.8 | 8.6 | 0.74 | 0.75 |
| 50 | 10 | 9.3 | 0.8 | 0.8 |
| 45 | 11 | 10.2 | 0.86 | 0.86 |
| 40 | 12.3 | 11.1 | 0.93 | 0.94 |
| 35 | 13.7 | 12.1 | 1.01 | 1.01 |
| 30 | 15.6 | 13.3 | 1.1 | 1.11 |
| 25 | 17.8 | 14.9 | 1.22 | 1.22 |
| 20 | 20.8 | 16.9 | 1.36 | 1.38 |
| 15 | 25.9 | 19.7 | 1.57 | 1.59 |
| 10 | 32.2 | 24.5 | 1.92 | 1.94 |
| 5 | 48.4 | 36.3 | 2.7 | 2.78 |

Table 2. Averaged percent of molecules of Set I identified in Top 1 to 10 with respect to different Gaussian standard deviation values (ppm) when ^13^C chemical shifts are used alone for identification.

| **Percentile (%)** | **Top 1** | **Top 2** | **Top 3** | **Top 4** | **Top 5** | **Top 6** | **Top 7** | **Top 8** | **Top 9** | **Top 10** |
| --- | --- | --- | --- | --- | --- | --- | --- | --- | --- | --- |
| 100 | 0 | 0.53 | 0.94 | 1.35 | 1.95 | 1.95 | 2 | 2.25 | 2.55 | 2.95 |
| 99.99 | 0 | 0.58 | 1.15 | 1.4 | 1.95 | 2.3 | 2.55 | 2.75 | 3 | 3.7 |
| 99.9 | 0.04 | 1.3 | 1.9 | 2.55 | 3 | 3.4 | 3.7 | 4.15 | 4.3 | 4.45 |
| 99 | 1.1 | 2.5 | 3.25 | 4 | 4.5 | 4.95 | 5.45 | 5.7 | 6.15 | 6.55 |
| 95 | 2.3 | 4 | 5 | 5.9 | 6.6 | 7.3 | 7.95 | 8.45 | 90.5 | 9.7 |
| 90 | 3.2 | 5.1 | 6.35 | 7.45 | 8.4 | 9.3 | 10 | 10.9 | 11.6 | 12.3 |
| 85 | 3.9 | 6.15 | 7.65 | 8.83 | 9.95 | 10.95 | 11.9 | 12.75 | 13.55 | 14.4 |
| 80 | 4.6 | 7.15 | 8.8 | 10.25 | 11.45 | 12.6 | 13.6 | 14.55 | 15.45 | 16.25 |
| 75 | 5.4 | 8.15 | 10 | 11.6 | 12.9 | 14.05 | 15.2 | 16.2 | 17.2 | 18.2 |
| 70 | 6.1 | 9.1 | 11.25 | 12.9 | 14.3 | 15.6 | 16.8 | 17.95 | 19.05 | 20.05 |
| 65 | 7 | 10.2 | 12.4 | 14.25 | 15.75 | 17.15 | 18.5 | 19.65 | 20.8 | 21.95 |
| 60 | 7.9 | 11.3 | 13.7 | 15.6 | 17.3 | 18.75 | 20.35 | 21.55 | 22.85 | 23.95 |
| 55 | 8.8 | 12.5 | 15.05 | 17.05 | 19 | 20.6 | 22.25 | 23.65 | 25 | 26.3 |
| 50 | 10 | 13.8 | 16.55 | 18.75 | 20.75 | 22.6 | 24.3 | 26 | 27.5 | 28.75 |

Table 3 Averaged percent of molecules of Set I identified in Top 1 to 10 with respect to different Gaussian standard deviation values (ppm) when ^1^H chemical shifts are used alone for identification.

| **Percentile (%)** | **Top 1** | **Top 2** | **Top 3** | **Top 4** | **Top 5** | **Top 6** | **Top 7** | **Top 8** | **Top 9** | **Top 10** |
| --- | --- | --- | --- | --- | --- | --- | --- | --- | --- | --- |
| 100 | 0 | 0.14 | 0.2 | 0.26 | 0.28 | 0.32 | 0.32 | 0.33 | 0.4 | 0.4 |
| 99.99 | - | 0.18 | 0.22 | 0.3 | 0.31 | 0.33 | 0.38 | 0.38 | 0.42 | 0.42 |
| 99.9 | 0.01 | 0.23 | 0.3 | 0.35 | 0.38 | 0.42 | 0.45 | 0.47 | 0.48 | 0.51 |
| 99 | 0.16 | 0.32 | 0.41 | 0.46 | 0.51 | 0.53 | 0.57 | 0.6 | 0.63 | 0.66 |
| 95 | 0.31 | 0.46 | 0.53 | 0.6 | 0.64 | 0.69 | 0.74 | 0.77 | 0.8 | 0.84 |
| 90 | 0.38 | 0.54 | 0.63 | 0.69 | 0.75 | 0.8 | 0.85 | 0.89 | 0.93 | 0.97 |
| 85 | 0.44 | 0.6 | 0.7 | 0.77 | 0.84 | 0.89 | 0.94 | 0.99 | 1.04 | 1.08 |
| 80 | 0.49 | 0.66 | 0.77 | 0.85 | 0.91 | 0.97 | 1.03 | 1.08 | 1.13 | 1.18 |
| 75 | 0.54 | 0.72 | 0.83 | 0.91 | 0.98 | 1.05 | 1.12 | 1.17 | 1.22 | 1.27 |
| 70 | 0.59 | 0.77 | 0.89 | 0.98 | 1.06 | 1.14 | 1.21 | 1.26 | 1.32 | 1.38 |
| 65 | 0.64 | 0.83 | 0.95 | 1.06 | 1.15 | 1.22 | 1.29 | 1.36 | 1.42 | 1.48 |
| 60 | 0.69 | 0.89 | 1.03 | 1.13 | 1.22 | 1.31 | 1.38 | 1.46 | 1.53 | 1.59 |
| 55 | 0.74 | 0.95 | 1.09 | 1.22 | 1.32 | 1.41 | 1.49 | 1.57 | 1.65 | 1.73 |
| 50 | 0.8 | 1.02 | 1.17 | 1.3 | 1.41 | 1.52 | 1.61 | 1.7 | 1.78 | 1.87 |

Table 4 Averaged percent of molecules of Set II identified in Top 1 to 10 with respect to different Gaussian standard deviation values (ppm) when ^13^C chemical shifts are used alone for identification.

| **Percentile (%)** | **Top 1** | **Top 2** | **Top 3** | **Top 4** | **Top 5** | **Top 6** | **Top 7** | **Top 8** | **Top 9** | **Top 10** |
| --- | --- | --- | --- | --- | --- | --- | --- | --- | --- | --- |
| 100 | 0 | 0.21 | 0.51 | 0.73 | 1.35 | 1.85 | 1.85 | 2.1 | 2.25 | 2.35 |
| 99.99 | - | 0.46 | 0.98 | 1.75 | 2.05 | 2.35 | 2.6 | 2.75 | 2.8 | 3.1 |
| 99.9 | 0.08 | 1.35 | 2.2 | 2.6 | 2.9 | 3.2 | 3.45 | 3.75 | 3.9 | 4.25 |
| 99 | 1.2 | 2.7 | 3.5 | 4.05 | 4.55 | 4.95 | 5.35 | 5.7 | 6.1 | 6.4 |
| 95 | 2.6 | 4.3 | 5.35 | 6.1 | 6.75 | 7.35 | 7.8 | 8.35 | 8.75 | 9.2 |
| 90 | 3.6 | 5.5 | 6.65 | 7.55 | 8.3 | 8.95 | 9.55 | 10.05 | 10.55 | 11.05 |
| 85 | 4.4 | 6.4 | 7.75 | 8.7 | 9.55 | 10.25 | 10.85 | 11.45 | 12 | 12.45 |
| 80 | 5.1 | 7.3 | 8.65 | 9.7 | 10.6 | 11.35 | 12.05 | 12.6 | 13.2 | 13.75 |
| 75 | 5.8 | 8.1 | 9.55 | 10.7 | 11.6 | 12.4 | 13.05 | 13.75 | 14.4 | 14.95 |
| 70 | 6.5 | 8.85 | 10.4 | 11.6 | 12.55 | 13.4 | 14.15 | 14.9 | 15.55 | 16.15 |
| 65 | 7.1 | 9.6 | 11.25 | 12.5 | 13.55 | 14.5 | 15.3 | 16 | 16.75 | 17.45 |
| 60 | 7.9 | 10.45 | 12.1 | 13.5 | 14.55 | 15.55 | 16.45 | 17.25 | 18 | 18.7 |
| 55 | 8.6 | 11.3 | 13.1 | 14.5 | 15.65 | 16.75 | 17.7 | 18.55 | 19.4 | 20.2 |
| 50 | 9.3 | 12.25 | 14.05 | 15.6 | 16.9 | 18.05 | 19.05 | 20.05 | 20.9 | 21.85 |

Table 5 Averaged percent of molecules of Set II identified in Top 1 to 10 with respect to different Gaussian standard deviation values (ppm) when ^1^H chemical shifts are used alone for identification.

| **Percentile (%)** | **Top 1** | **Top 2** | **Top 3** | **Top 4** | **Top 5** | **Top 6** | **Top 7** | **Top 8** | **Top 9** | **Top 10** |
| --- | --- | --- | --- | --- | --- | --- | --- | --- | --- | --- |
| 100 | - | 0.02 | 0.03 | 0.03 | 0.09 | 0.1 | 0.13 | 0.13 | 0.16 | 0.18 |
| 99.99 | - | 0.03 | 0.04 | 0.08 | 0.12 | 0.14 | 0.16 | 0.18 | 0.2 | 0.2 |
| 99.9 | 0.01 | 0.08 | 0.11 | 0.14 | 0.17 | 0.19 | 0.22 | 0.23 | 0.25 | 0.26 |
| 99 | 0.17 | 0.16 | 0.19 | 0.24 | 0.36 | 0.28 | 0.31 | 0.32 | 0.34 | 0.35 |
| 95 | 0.31 | 0.25 | 0.3 | 0.34 | 0.37 | 0.39 | 0.41 | 0.43 | 0.45 | 0.47 |
| 90 | 0.38 | 0.31 | 0.37 | 0.41 | 0.44 | 0.46 | 0.49 | 0.51 | 0.53 | 0.55 |
| 85 | 0.45 | 0.36 | 0.42 | 0.46 | 0.49 | 0.52 | 0.55 | 0.57 | 0.6 | 0.61 |
| 80 | 0.5 | 0.4 | 0.46 | 0.51 | 0.64 | 0.57 | 0.61 | 0.63 | 0.65 | 0.68 |
| 75 | 0.55 | 0.44 | 0.5 | 0.55 | 0.59 | 0.62 | 0.66 | 0.69 | 0.71 | 0.74 |
| 70 | 0.59 | 0.48 | 0.54 | 0.59 | 0.64 | 0.68 | 0.71 | 0.74 | 0.77 | 0.8 |
| 65 | 0.64 | 0.52 | 0.59 | 0.64 | 0.69 | 0.73 | 0.76 | 0.8 | 0.83 | 0.86 |
| 60 | 0.69 | 0.55 | 0.63 | 0.69 | 0.74 | 0.78 | 0.82 | 0.86 | 0.89 | 0.92 |
| 55 | 0.75 | 0.6 | 0.68 | 0.74 | 0.79 | 0.84 | 0.88 | 0.92 | 0.95 | 0.99 |
| 50 | 0.8 | 0.64 | 0.73 | 0.79 | 0.85 | 0.9 | 0.95 | 0.99 | 1.03 | 1.07 |


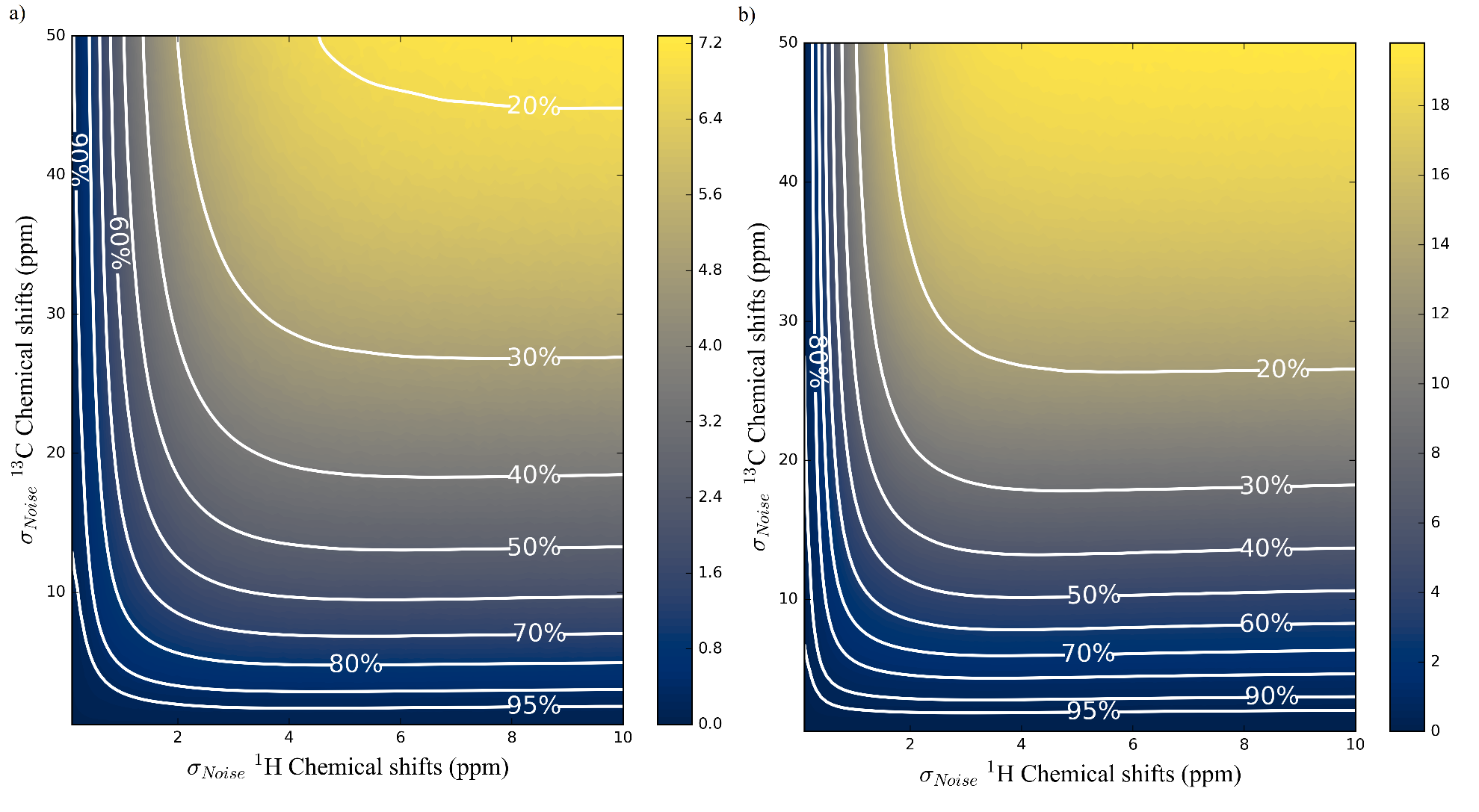


Figure 1 Standard deviations of ranks with respect to the carbon and proton errors and contour lines for the different level of identification ratios when carbons and protons are used together for a-b) Set I (water soluble molecules) and c-d) Set II (chloroform soluble molecules)

The relationship (i.e. trade-off) between ^13^C and ^1^H chemical shift errors given by the white contour lines are predicted by using Eqn S1, Eqn S2, and Eqn S3 as showed by red dashed lines in Figure 1 and Figure 2 for Set I and Set II, respectively.

|  | $\mathrm{MAE}\left( 13C \right)=a+b\exp^{(-cx)}$ | Eqn S1 |
| --- | --- | --- |
|  | $\mathrm{MAE}\left( 13C \right)=a/\left( \mathrm{MAE}\left( 1H \right)-b \right)+c$ | Eqn S2 |
|  | $\mathrm{MAE}\left( 13C \right)=a+b\exp^{(-cx)}+d\exp^{(-ex)}$ | Eqn S3 |

The fitting parameters predicted for Eqn S1, Eqn S2, and Eqn S3 for Set I and Set II are given in Table 6-8, respectively along with Coefficient of Determination (R^2^) and Sum of Squared Difference (SSD) values.

| **% identification** | **Set I** | | | | | **Set II** | | | | |
| --- | --- | --- | --- | --- | --- | --- | --- | --- | --- | --- |
|  | **R2** | **SSD** | **a** | **b** | **c** | **R2** | **SSD** | **a** | **b** | **c** |
| 95 | 0.99 | 17.84 | 68.20 | 7.55 | 2.35 | 1.00 | 0.27 | 7.80 | 4.92 | 1.90 |
| 90 | 0.99 | 17.38 | 104.49 | 5.66 | 3.42 | 1.00 | 6.47 | 47.63 | 8.11 | 3.02 |
| 85 | 0.99 | 15.06 | 106.06 | 4.33 | 4.34 | 0.99 | 12.98 | 103.02 | 7.81 | 3.92 |
| 80 | 0.99 | 11.26 | 99.12 | 3.42 | 5.24 | 0.99 | 14.21 | 117.15 | 6.42 | 4.71 |
| 75 | 0.99 | 7.44 | 89.37 | 2.76 | 6.18 | 0.99 | 12.42 | 110.30 | 5.20 | 5.50 |
| 70 | 1.00 | 4.53 | 83.65 | 2.30 | 7.22 | 0.99 | 10.15 | 98.81 | 4.25 | 6.31 |
| 65 | 1.00 | 2.39 | 81.60 | 1.99 | 8.40 | 0.99 | 7.70 | 87.77 | 3.53 | 7.17 |
| 60 | 1.00 | 1.30 | 81.41 | 1.75 | 9.75 | 0.99 | 5.43 | 78.92 | 2.98 | 8.10 |
| 55 | 1.00 | 0.49 | 81.64 | 1.55 | 11.29 | 0.99 | 3.37 | 72.19 | 2.55 | 9.12 |
| 50 | 1.00 | 0.16 | 86.41 | 1.41 | 13.17 | 1.00 | 1.71 | 67.64 | 2.23 | 10.29 |

| **% identification** | **Set I** | | | | | **Set II** | | | | |
| --- | --- | --- | --- | --- | --- | --- | --- | --- | --- | --- |
|  | **R2** | **SSD** | **a** | **b** | **c** | **R2** | **SSD** | **a** | **b** | **c** |
| 95 | 1.00 | 0.81 | 1.33 | 0.12 | 1.43 | 1.00 | 2.23 | 0.35 | 0.11 | 1.83 |
| 90 | 1.00 | 1.13 | 1.95 | 0.23 | 2.21 | 1.00 | 1.49 | 0.66 | 0.16 | 2.52 |
| 85 | 1.00 | 0.55 | 2.50 | 0.31 | 2.97 | 1.00 | 1.00 | 1.01 | 0.20 | 3.16 |
| 80 | 1.00 | 0.46 | 3.16 | 0.38 | 3.68 | 1.00 | 0.55 | 1.36 | 0.24 | 3.79 |
| 75 | 1.00 | 0.42 | 3.92 | 0.45 | 4.45 | 1.00 | 0.83 | 1.80 | 0.28 | 4.40 |
| 70 | 1.00 | 0.39 | 4.71 | 0.52 | 5.31 | 1.00 | 0.73 | 2.20 | 0.32 | 5.10 |
| 65 | 1.00 | 0.32 | 5.44 | 0.61 | 6.34 | 1.00 | 0.58 | 2.62 | 0.36 | 5.84 |
| 60 | 1.00 | 0.21 | 6.03 | 0.73 | 7.61 | 1.00 | 0.58 | 3.02 | 0.41 | 6.69 |
| 55 | 1.00 | 0.15 | 6.64 | 0.86 | 9.09 | 1.00 | 0.60 | 3.49 | 0.46 | 7.62 |
| 50 | 1.00 | 0.09 | 6.51 | 1.08 | 11.10 | 1.00 | 0.62 | 3.88 | 0.53 | 8.71 |

Table 8 Fitting parameters predicted for Set I and Set II using Eqn S3

| **Percentages**  **(%)** | **Set I** | | | | | | | **Set II** | | | | | | |
| --- | --- | --- | --- | --- | --- | --- | --- | --- | --- | --- | --- | --- | --- | --- |
|  | **R^2^** | **SSD** | **a** | **b** | **c** | **d** | **e** | **R^2^** | **SSD** | **a** | **b** | **c** | **d** | **e** |
| 95 | 1.00 | 1.05 | 8.51 | 2.32 | 134.40 | 12.85 | 1.98 | 1.00 | 0.27 | 3.90 | 4.92 | 3.90 | 4.92 | 1.90 |
| 90 | 1.00 | 0.30 | 12.45 | 2.02 | 425.88 | 10.98 | 3.00 | 1.00 | 1.00 | 4.08 | 2.12 | 56.18 | 10.62 | 2.78 |
| 85 | 1.00 | 0.21 | 15.57 | 1.78 | 628.58 | 9.26 | 3.88 | 1.00 | 0.53 | 10.58 | 2.67 | 329.56 | 13.97 | 3.61 |
| 80 | 1.00 | 0.15 | 18.26 | 1.60 | 656.57 | 7.70 | 4.77 | 1.00 | 0.46 | 13.78 | 2.45 | 713.93 | 13.24 | 4.38 |
| 75 | 1.00 | 0.09 | 22.25 | 1.49 | 713.67 | 6.73 | 5.73 | 1.00 | 0.28 | 14.90 | 2.13 | 781.78 | 11.21 | 5.15 |
| 70 | 1.00 | 0.09 | 26.53 | 1.40 | 804.17 | 6.03 | 6.80 | 1.00 | 0.25 | 16.05 | 1.90 | 726.53 | 9.50 | 5.95 |
| 65 | 1.00 | 0.08 | 33.73 | 1.38 | 1424.80 | 5.95 | 8.03 | 0.99 | 7.70 | -17.24 | 3.53 | 105.00 | 3.53 | 7.17 |
| 60 | 1.00 | 0.07 | 41.99 | 1.33 | 7848.66 | 6.72 | 9.43 | 1.00 | 0.18 | 22.11 | 1.73 | 1073.85 | 8.14 | 7.79 |
| 55 | 1.00 | 0.05 | 55.06 | 1.33 | 1.16E+06 | 9.45 | 11.08 | 1.00 | 0.13 | 26.11 | 1.67 | 1456.72 | 7.75 | 8.86 |
| 50 | 1.00 | 0.01 | 70.07 | 1.31 | 4.30E+18 | 23.88 | 13.06 | 1.00 | 0.05 | 31.91 | 1.64 | 3212.26 | 7.92 | 10.08 |


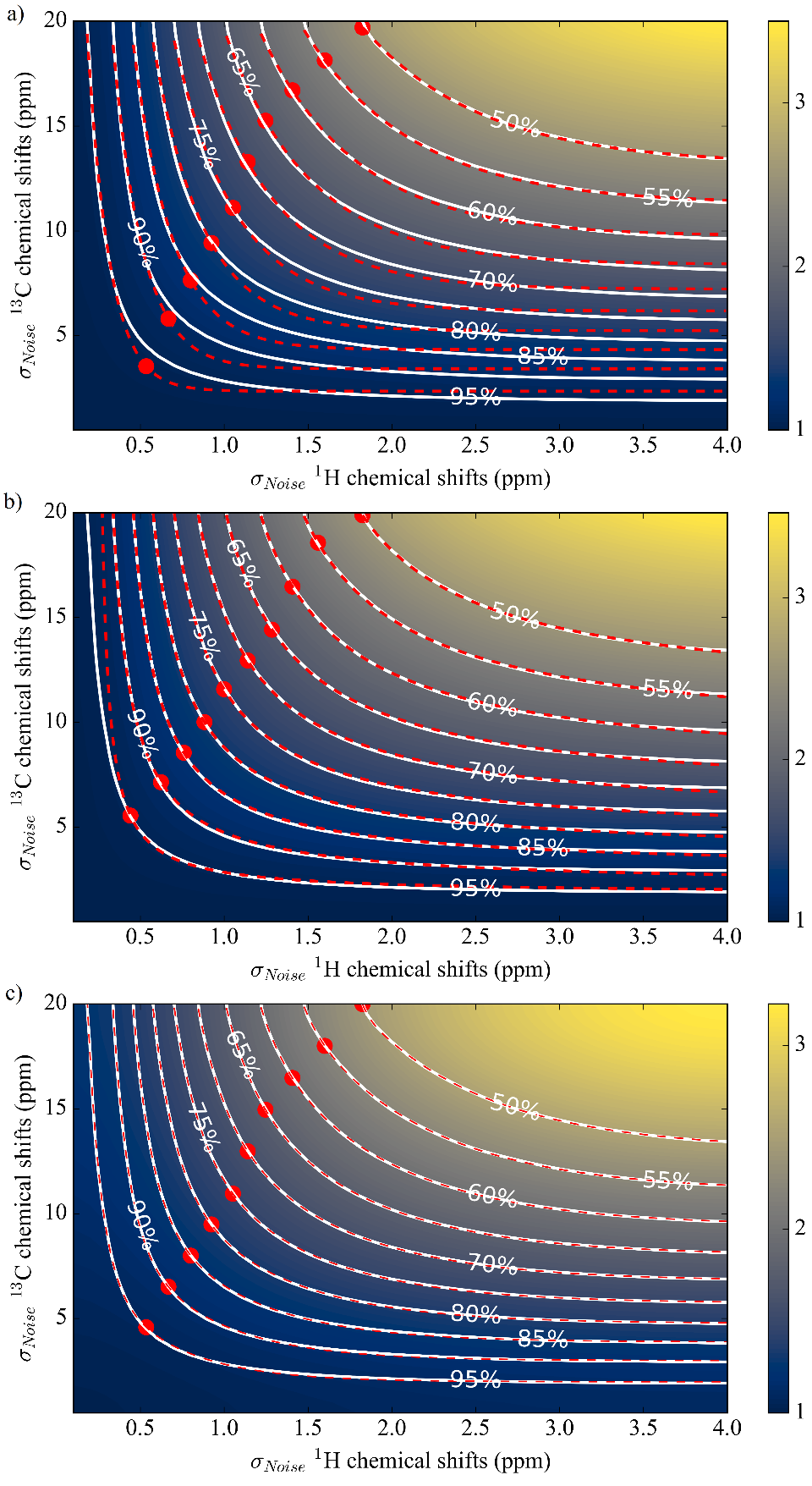


Figure 2 Mean of ranks changing with the ^13^C and ^1^H NMR chemical shifts errors when used together for identification for Set I using a) Eqn S1, b) Eqn S2, and c) Eqn S3. White (actual data) and red dashed (predicted data) contour lines represent the same level of identification with changing errors with respect to one another.


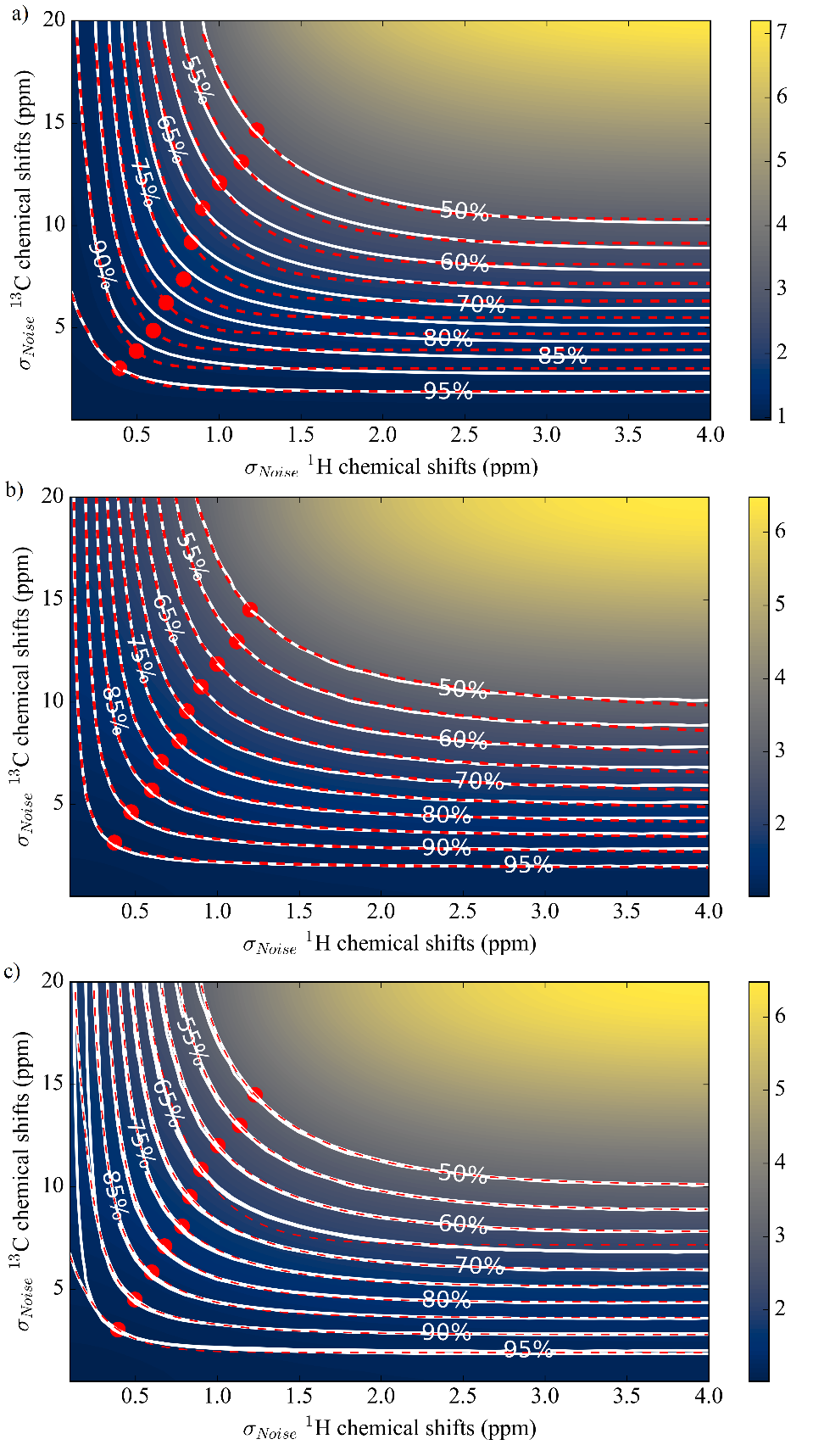


Figure 3 Mean of ranks changing with the ^13^C and ^1^H NMR chemical shifts errors when used together for identification for Set II using a) Eqn S1, b) Eqn S2, and c) Eqn S3. White (actual data) and red dashed (predicted data) contour lines represent the same level of identification with changing errors with respect to one another.

Table 9 Optimum MAEs at different Gaussian standard deviation (σ) values (ppm) for Set I and Set II when ^13^C and ^1^H NMR chemical shifts are used alone and together for identification

| **Percentages**  **(%)** | **σ (ppm) - Set I** | | | | **σ (ppm) - Set II** | | | |
| --- | --- | --- | --- | --- | --- | --- | --- | --- |
|  | **^13^C alone** | **^1^H alone** | **Both ^13^C & ^1^H** | | **^13^C alone** | **^1^H alone** | **Both ^13^C & ^1^H** | |
| 100 | 0 | 0 | 0 | 0 | 0 | 0 | 0 | 0 |
| 99.99 | 0.002 | - |  |  | - |  |  |  |
| 99.9 | 0.04 | 0.01 | - | - | 0.08 | 0.01 | - | - |
| 99 | 1.1 | 0.16 | - | - | 1.2 | 0.17 | - | - |
| 95 | 2.3 | 0.31 | 4.41 | 0.73 | 2.6 | 0.31 | 3.13 | 0.37 |
| 90 | 3.2 | 0.38 | 6.12 | 0.85 | 3.6 | 0.38 | 4.57 | 0.48 |
| 85 | 3.9 | 0.44 | 7.57 | 0.97 | 4.4 | 0.45 | 5.76 | 0.59 |
| 80 | 4.6 | 0.49 | 9.09 | 1.07 | 5.1 | 0.5 | 6.92 | 0.68 |
| 75 | 5.4 | 0.54 | 10.73 | 1.19 | 5.8 | 0.55 | 8.09 | 0.77 |
| 70 | 6.1 | 0.59 | 12.34 | 1.30 | 6.5 | 0.59 | 9.30 | 0.84 |
| 65 | 7 | 0.64 | 14.23 | 1.42 | 7.1 | 0.64 | 10.41 | 0.94 |
| 60 | 7.9 | 0.69 | 16.28 | 1.56 | 7.9 | 0.69 | 11.68 | 1.02 |
| 55 | 8.8 | 0.74 | 18.55 | 1.71 | 8.6 | 0.75 | 12.90 | 1.13 |
| 50 | 10 | 0.8 | 21.41 | 0.60 | 9.3 | 0.8 | 14.27 | 1.23 |

# **
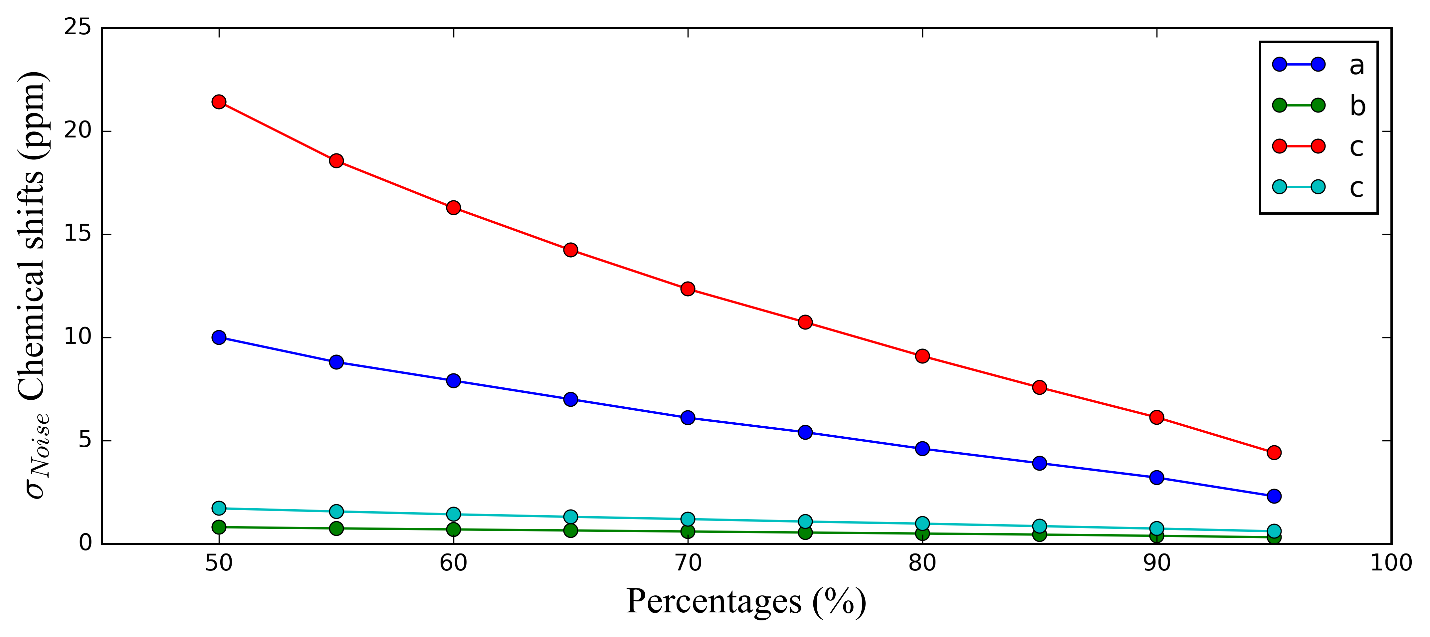
**

Figure 4 Optimum points obtained when a) ^13^C alone, b) ^1^H alone, c) ^13^C together, and d) ^1^H together are used with respect to level of identifications for Set I


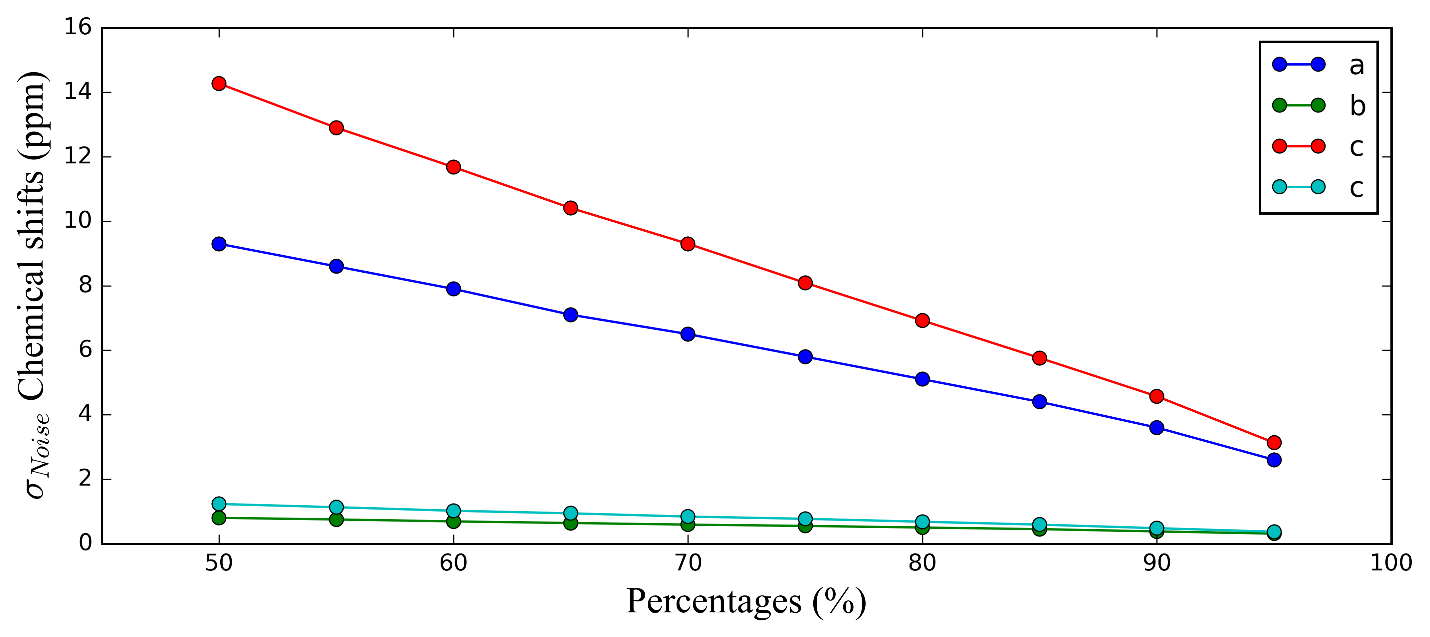


Figure 5 Optimum points obtained when a) ^13^C alone, b) ^1^H alone, c) ^13^C together, and d) ^1^H together are used with respect to level of identifications for Set II

# **S2. Results of Case II**


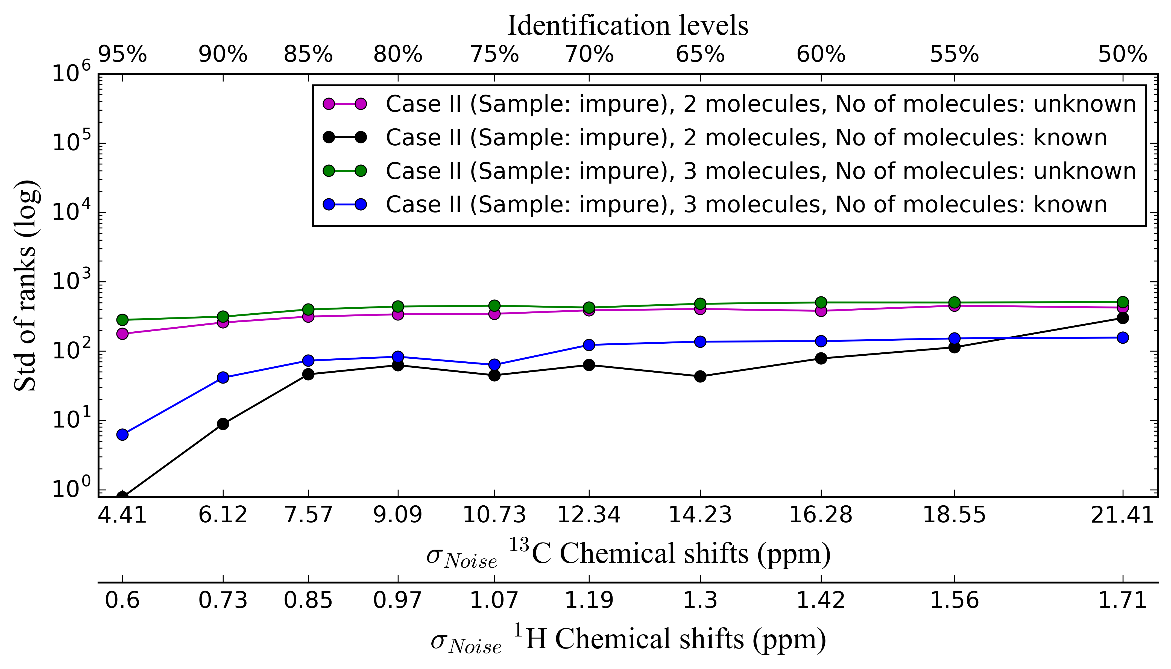


Figure 6 Standard deviation of ranks (at logarithmic scale) of Case II for pairs and triplets when number of molecules in samples are known and unknown at optimum points for Set I


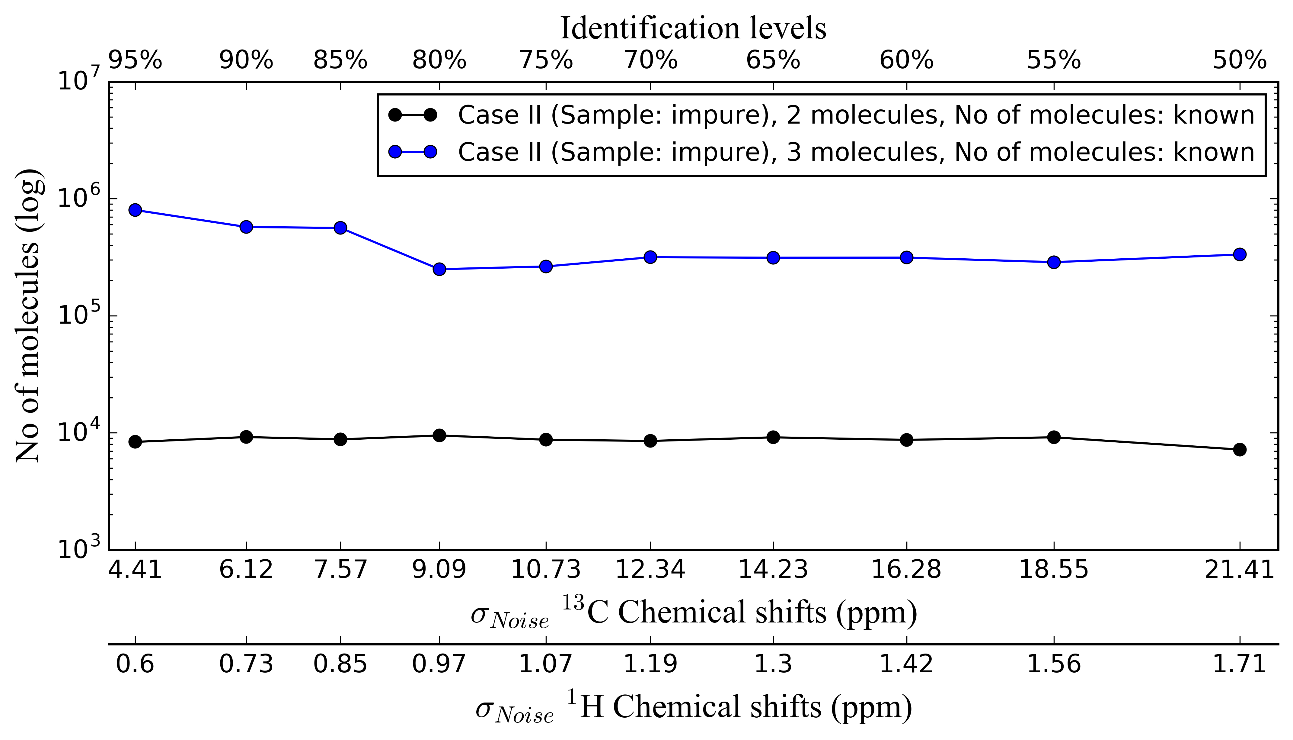


Figure 7 Average number of molecules (at logarithmic scale) used to be matched with the molecule set of interest in runs of Case II for pairs and triplets when number of molecules in samples are known at optimum points for Set I


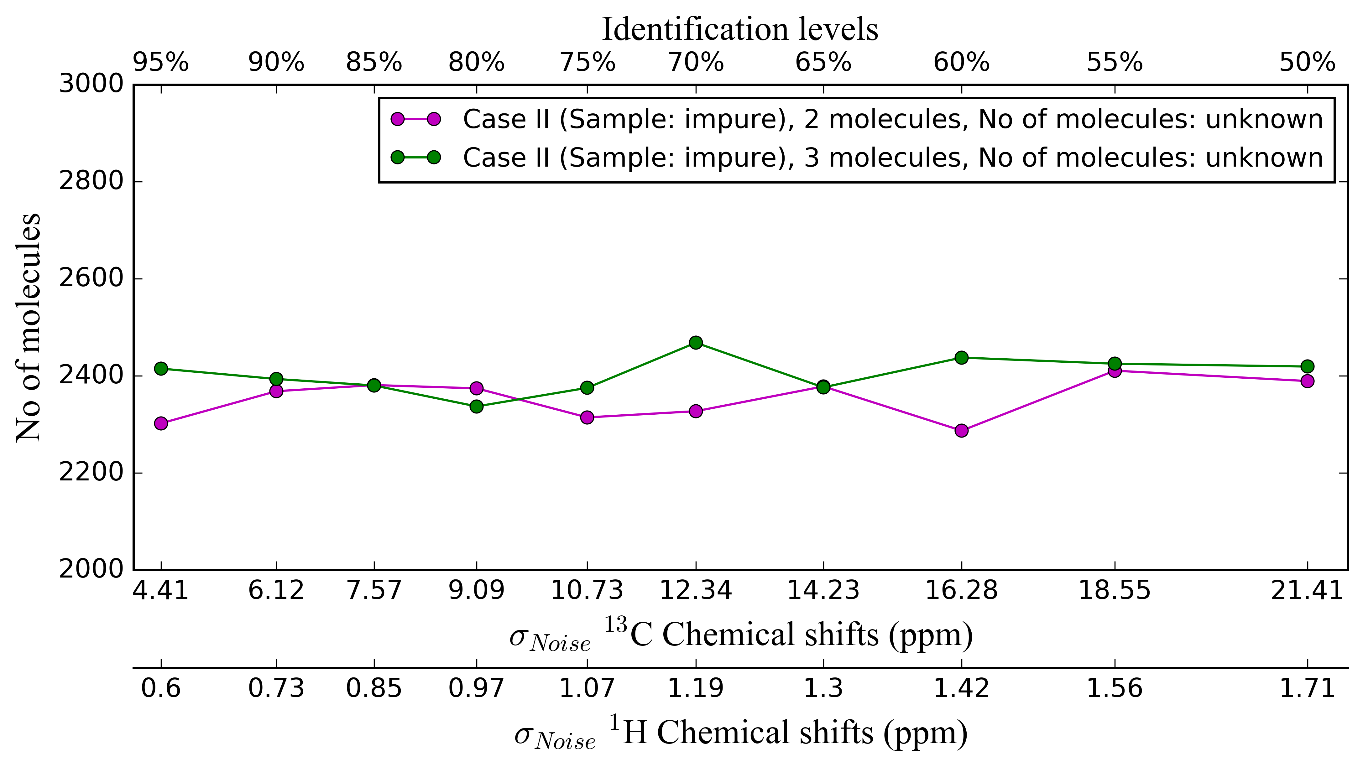


Figure 8 Average number of molecules (at logarithmic scale) used to be matched with the molecule set of interest in runs of Case II for pairs and triplets when number of molecules in samples are unknown at optimum points for Set I


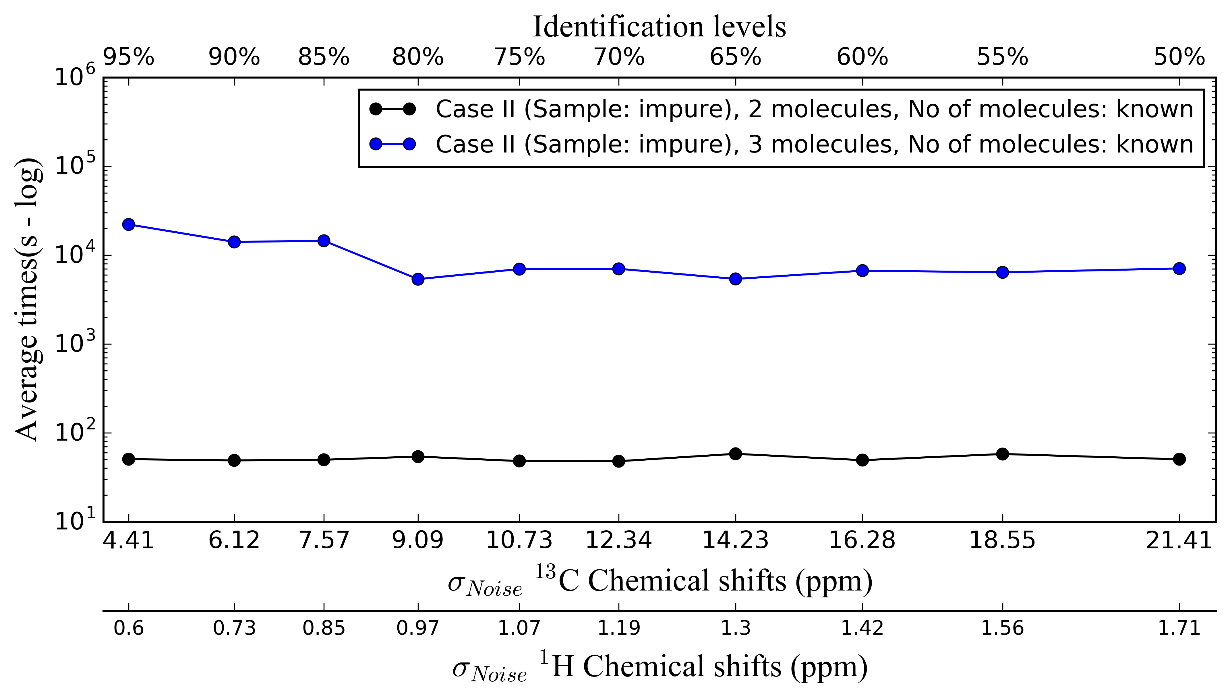


Figure 9 Average computational times (s) (at logarithmic scale) spent for runs of Case II for pairs and triplets when number of molecules in samples are known at optimum points for Set I


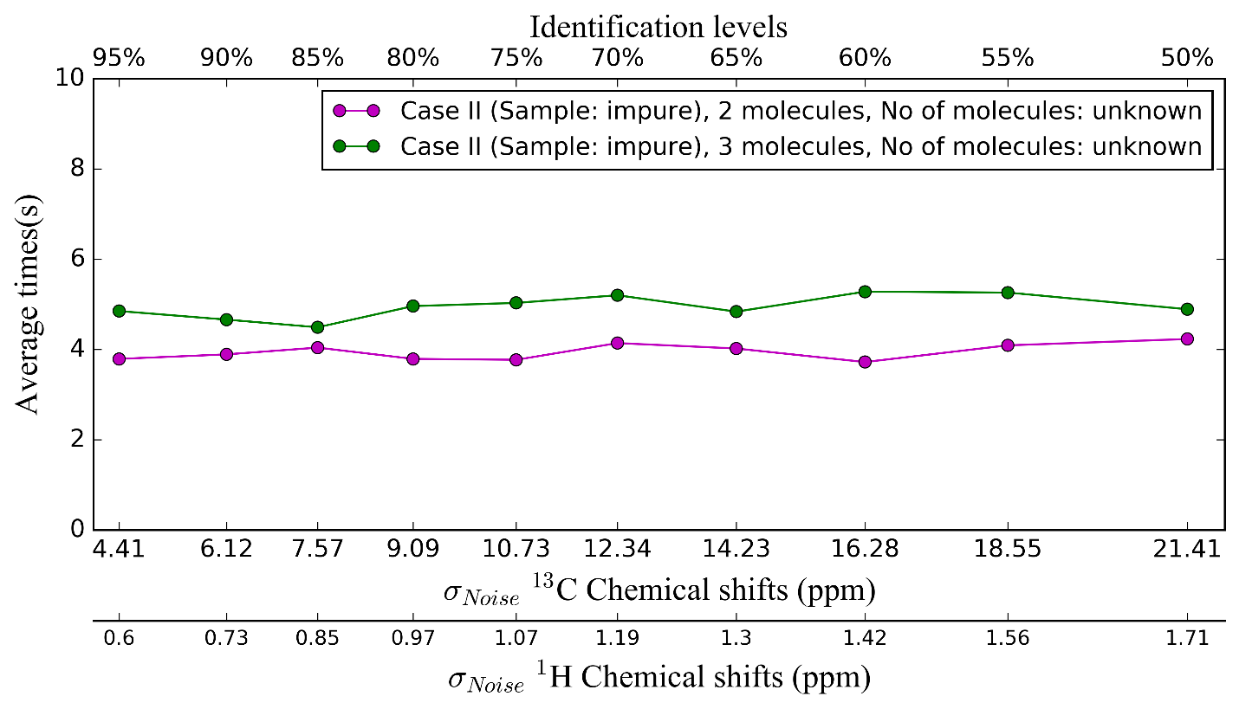


Figure 10 Average computational times (s) (at logarithmic scale) spent for runs of Case II for pairs and triplets when number of molecules in samples are unknown at optimum points for Set I

# **S3. Results of the comparisons of different DFT methods over NMR spectrum**

Isotropic shielding values are compared using ISiCLE for the following 3 different following DFT methods:

- Method 1: B3LYP/cc-pVDZ//B3LYP/3-21G
- Method 2: B3LYP/cc-pVDZ//B3LYP/6-31G*
- Method 3: B3LYP/cc-pVTZ//B3LYP/6-31G*

The comparisons are performed over randomly chosen 5 molecules from Set I (water-solvated molecules) and Set II (chloroform-solvated molecules). Molecules are in different shapes and sizes. The images of the molecules taken from Set I and Set II are given in Figure 11 and Figure 12, and the details of the molecules are given in Table 10 and Table 11, respectively.


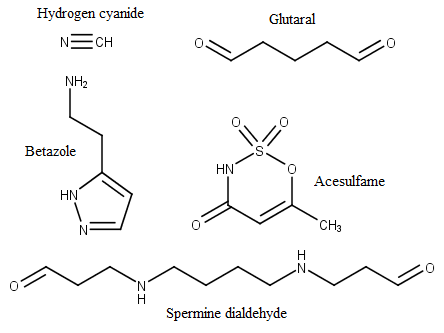


Figure 11 Molecules chosen from the Set I (water-solvated)


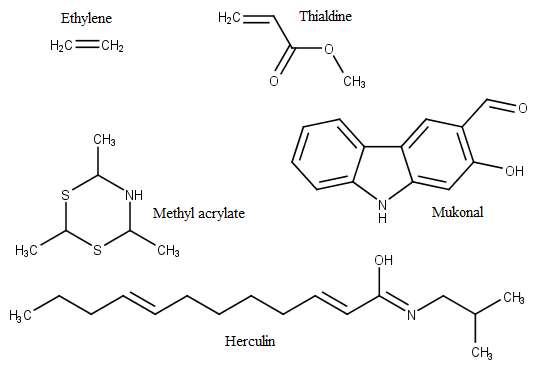


Figure 12 Molecules chosen from the Set II (chloroform-solvated)

Table 10 Features of the 5 molecules randomly chosen from the Set I (water-solvated)

| **Common Name** | Hydrogen cyanide | Glutaral | Betazole | Acesulfame | Spermine dialdehyde |
| --- | --- | --- | --- | --- | --- |
| **IUPAC Name** | Formonitrile | Pentanedial | 2-(1H-pyrazol-5-yl)ethan-1-amine | 6-methyl-3,4-dihydro-1,2,3-oxathiazine-2,2,4-trione | 3-4-3-oxopropylamino]butyl}amino)propanal |
| **InChI** | InChI=1S/CHN/c1-2/h1H | InChI=1S/C5H8O2/c6-4-2-1-3-5-7/h4-5H,1-3H2 | InChI=1S/C5H9N3/c6-3-1-5-2-4-7-8-5/h2,4H,1,3,6H2,(H,7,8) | InChI=1S/C4H5NO4S/c1-3-2-4(6)5-10(7,8)9-3/h2H,1H3,(H,5,6) | InChI=1S/C10H20N2O2/c13-9-3-7-11-5-1-2-6-12-8-4-10-14/h9-12H,1-8H2 |
| **InChIKey** | LELOWRISYMNNSU-UHFFFAOYSA-N | SXRSQZLOMIGNAQ-UHFFFAOYSA-N | JXDFEQONERDKSS-UHFFFAOYSA-N | YGCFIWIQZPHFLU-UHFFFAOYSA-N | WPBJCXUUUSDQJO-UHFFFAOYSA-N |
| **SMILES** | C#N | O=CCCCC=O | NCCC1=CC=NN1 | CC1=CC(=O)NS(=O)(=O)O1 | O=CCCNCCCCNCCC=O |
| **Chemical Formula** | CHN | C5H8O2 | C5H9N3 | C4H5NO4S | C10H20N2O2 |
| **Molecular Weight** | 27.0253 | 100.1158 | 111.1451 | 163.152 | 200.278 |
| **LogP** | -0.65 | 0.93 | -0.64 | -0.1 | 0.45 |
| **LogS** | -0.92 | -0.19 | 0.15 | -1.15 | -2.39 |
| **Solubility** | 3.26 g/L | 64 g/L | 156 g/L | 11.4 g/L | 0.81 g/L |
| **LogP** | -0.35 | -0.27 | -0.62 | -0.55 | -0.87 |
| **Pka (Acidic)** | 9.5 | 14.48 | 14.52 | 3.02 | 13.85 |
| **Pka (Basic)** |  | -6.6 | 9.79 | -6 | 10.23 |
| **Rotatable Bond Count** | 0 | 4 | 2 | 0 | 11 |
| **Direct Parent** | Nitriles | Alpha-hydrogen aldehydes | 2-arylethylamines | Organic sulfuric acids and derivatives | Alpha-hydrogen aldehydes |
| **Kingdom Class** | Chemical entities | Organic compounds | Chemical entities | Chemical entities | Chemical entities |
| **Super Class** | Organic compounds | Organic oxygen compounds | Organic compounds | Organic compounds | Organic compounds |
| **Class** | Organic nitrogen compounds | Organooxygen compounds | Organic nitrogen compounds | Organic acids and derivatives | Organic oxygen compounds |
| **Sub Class** | Organonitrogen compounds | Carbonyl compounds | Organonitrogen compounds | Organic sulfuric acids and derivatives | Organooxygen compounds |
| **Harary Index** | 1 | 11.15 | 15.57 | 23.42 | 31.52 |
| **Balaban Index** | 1 | 2.45 | 2.06 | 2.51 | 2.78 |
| **Ring Bond %** | 0 | 0 | 0.294118 | 0.4 | 0 |

Table 11 Features of the 5 molecules randomly chosen from the Set II (chloroform-solvated)

| **Common Name** | Ethylene | Methyl acrylate | Thialdine | Mukonal | Herculin |
| --- | --- | --- | --- | --- | --- |
| **IUPAC Name** | ethene | methyl prop-2-enoate | 2,4,6-trimethyl-1,3,5-dithiazinane | 2-hydroxy-9H-carbazole-3-carbaldehyde | (Z,2E,8E)-N-(2-methylpropyl)dodeca-2,8-dienimidic acid |
| **InChI** | InChI=1S/C2H4/c1-2/h1-2H2 | InChI=1S/C4H6O2/c1-3-4(5)6-2/h3H,1H2,2H3 | InChI=1S/C6H13NS2/c1-4-7-5(2)9-6(3)8-4/h4-7H,1-3H3 | InChI=1S/C13H9NO2/c15-7-8-5-10-9-3-1-2-4-11(9)14-12(10)6-13(8)16/h1-7,14,16H | InChI=1S/C16H29NO/c1-4-5-6-7-8-9-10-11-12-13-16(18)17-14-15(2)3/h6-7,12-13,15H,4-5,8-11,14H2,1-3H3,(H,17,18)/b7-6+,13-12+ |
| **InChIKey** | VGGSQFUCUMXWEO-UHFFFAOYSA-N | BAPJBEWLBFYGME-UHFFFAOYSA-N | FBMVFHKKLDGLJA-UHFFFAOYSA-N | NEAHWGSQUXSRNW-UHFFFAOYSA-N | JNPRQUIWDVDHIT-GYIPPJPDSA-N |
| **SMILES** | C=C | COC(=O)C=C | CC1NC(C)SC(C)S1 | OC1=C(C=O)C=C2C(NC3=CC=CC=C23)=C1 | CCC\C=C\CCCC\C=C\C(\O)=N\CC(C)C |
| **Chemical Formula** | C2H4 | C4H6O2 | C6H13NS2 | C13H9NO2 | C16H29NO |
| **Molecular Weight** | 28.0532 | 86.0892 | 163.304 | 211.2161 | 251.4076 |
| **LogP** | 0.9 | 0.67 | 1.3 | 2.86 | 5.85 |
| **LogS** | -0.94 | -0.13 | -1.72 | -3.47 | -5.47 |
| **Solubility** | 3.25 g/L | 64.2 g/L | 3.12 g/L | 0.072 g/L | 8.500E-04 g/L |
| **LogP** | 1.11 | 0.91 | 1.39 | 3.15 | 5.59 |
| **Pka (Acidic)** |  |  |  | 8.07 | 8.69 |
| **Pka (Basic)** |  | -6.8 | 5.87 | -6 | 6.02 |
| **Rotatable Bond Count** | 0 | 2 | 0 | 1 | 10 |
| **Direct Parent** | Acyclic olefins | Enoate esters | 1,3,5-dithiazinanes | Carbazoles | N-acyl amines |
| **Kingdom Class** | Organic compounds | Organic compounds | Chemical entities | Chemical entities | Chemical entities |
| **Super Class** | Hydrocarbons | Organic acids and derivatives | Organic compounds | Organic compounds | Organic compounds |
| **Class** | Olefins | Carboxylic acids and derivatives | Organoheterocyclic compounds | Organoheterocyclic compounds | Lipids and lipid-like molecules |
| **Sub Class** | Acyclic olefins | Carboxylic acid derivatives | Azacyclic compounds | Indoles and derivatives | Fatty Acyls |
| **Harary Index** | 1 | 9.08 | 19.5 | 49.97 | 46.5 |
| **Balaban Index** | 1 | 2.75 | 2.08 | 1.54 | 3.1 |
| **Ring Bond %** | 0 | 0 | 0.272727 | 0.555556 | 0 |


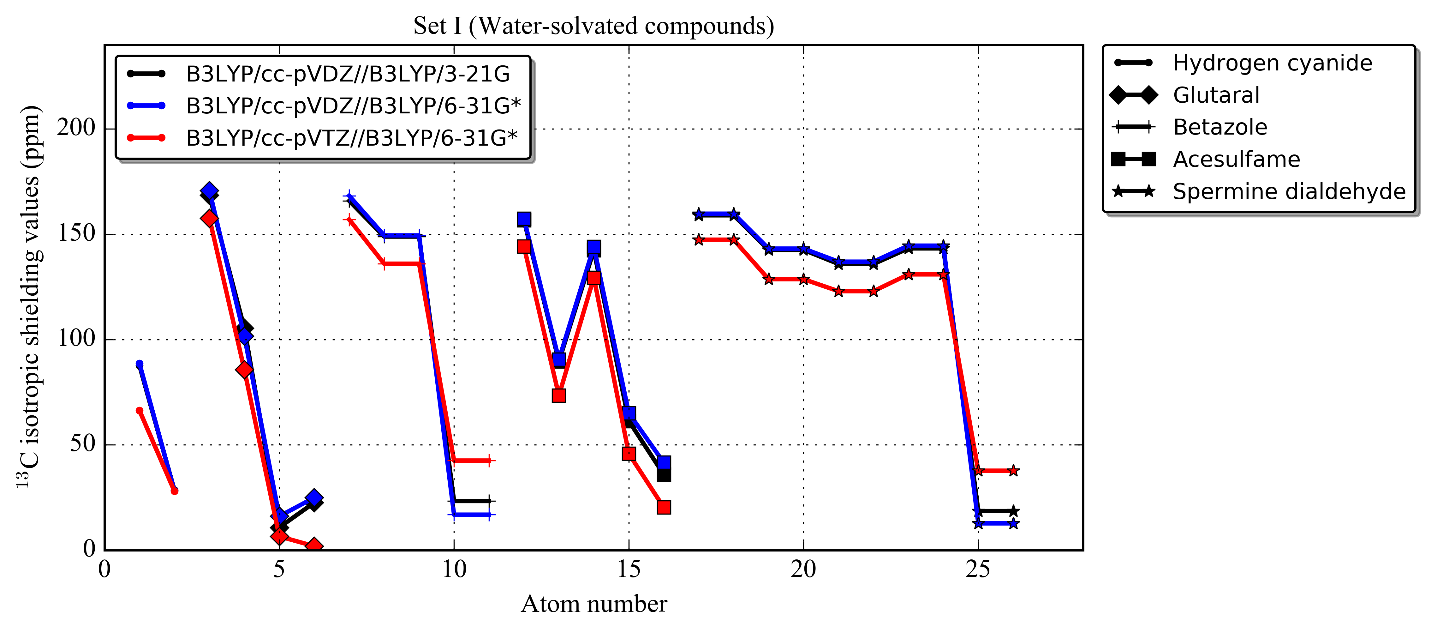


Figure 13 ^13^C isotropic shielding values of the water-solvated molecules (Set I), hydrogen cyanide, glutaral, betazole, acesulfame, spermine dialdehyde, for the DFT methods of B3LYP/cc-pVDZ//B3LYP/3-21G (black), B3LYP/cc-pVDZ//B3LYP/6-31G* (blue), B3LYP/cc-pVTZ//B3LYP/6-31G* (red)


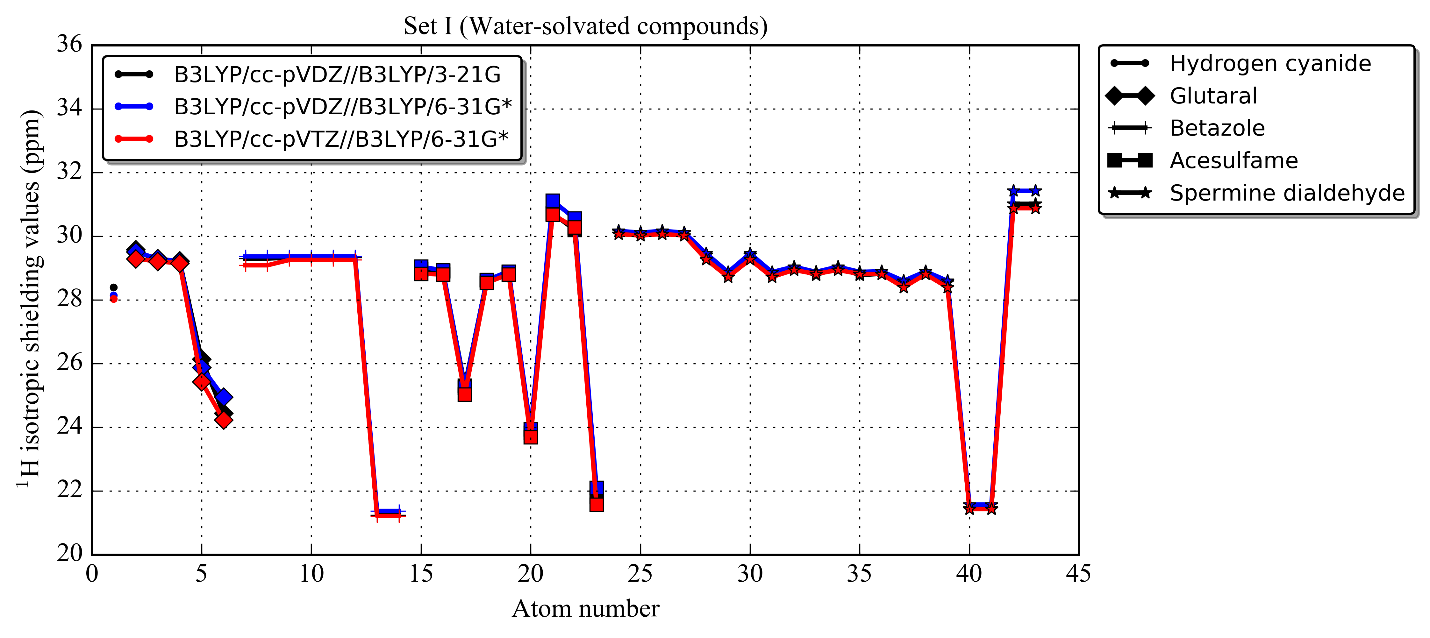


Figure 14 ^1^H isotropic shielding values of the water-solvated molecules (Set I), hydrogen cyanide, glutaral, betazole, acesulfame, spermine dialdehyde, for the DFT methods of B3LYP/cc-pVDZ//B3LYP/3-21G (black), B3LYP/cc-pVDZ//B3LYP/6-31G* (blue), B3LYP/cc-pVTZ//B3LYP/6-31G* (red)


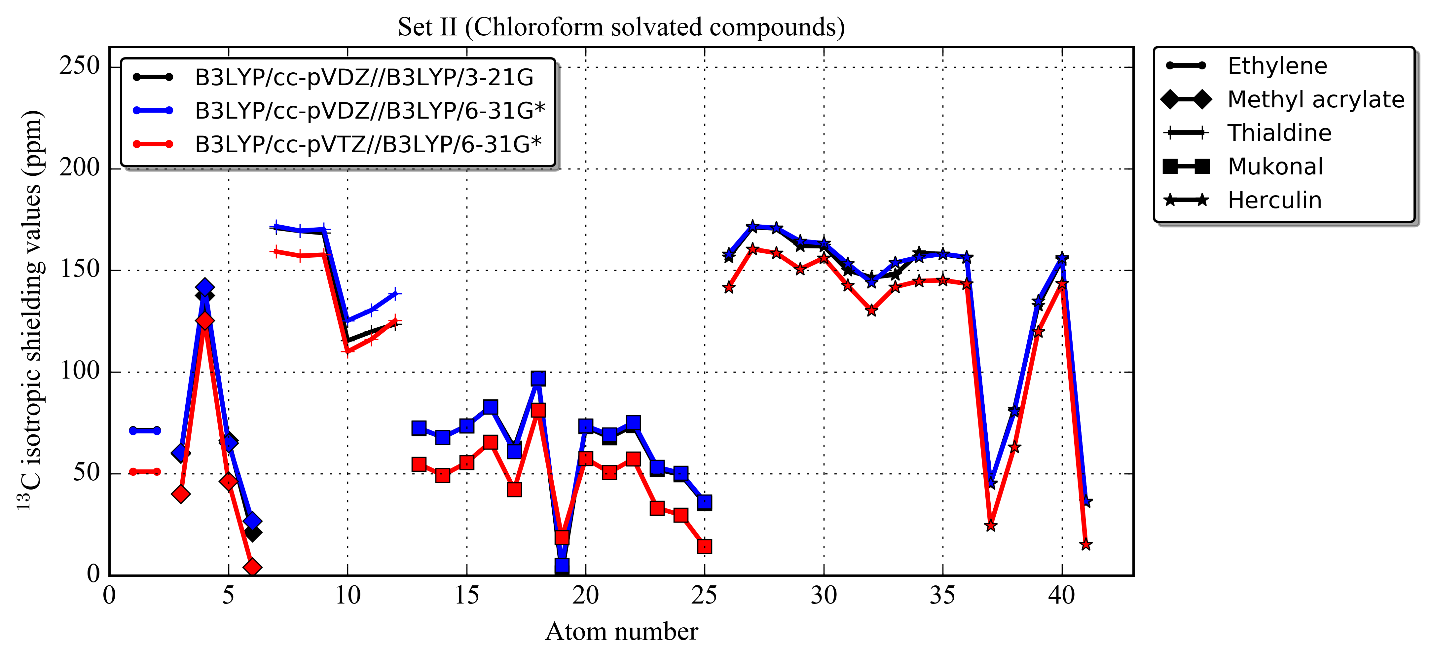


Figure 15 ^13^C isotropic shielding values of the chloroform-solvated molecules (Set II), ethylene, methyl acrylate, thialdine, Mukonal, herculin, for the DFT methods of B3LYP/cc-pVDZ//B3LYP/3-21G (black), B3LYP/cc-pVDZ//B3LYP/6-31G* (blue), B3LYP/cc-pVTZ//B3LYP/6-31G* (red)


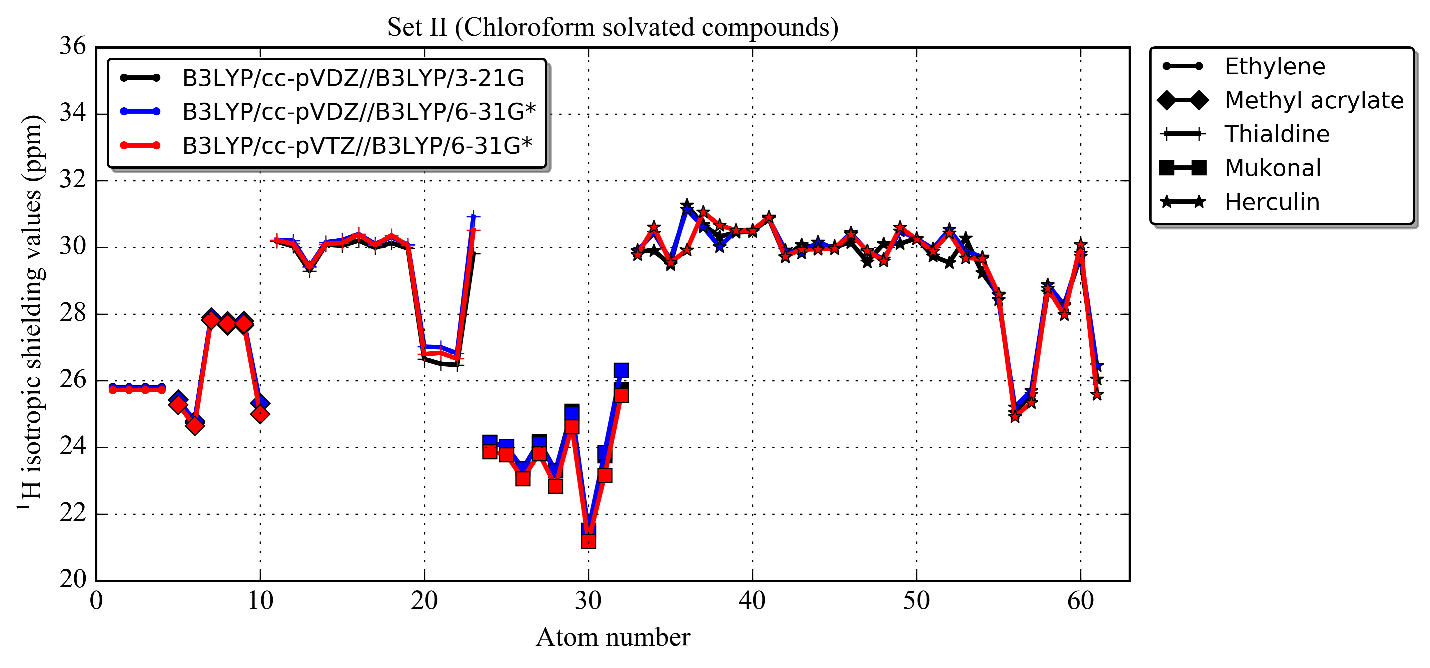


Figure 16 ^1^H isotropic shielding values of the chloroform-solvated molecules (Set II), ethylene, methyl acrylate, thialdine, Mukonal, herculin, for the DFT methods of B3LYP/cc-pVDZ//B3LYP/3-21G (black), B3LYP/cc-pVDZ//B3LYP/6-31G* (blue), B3LYP/cc-pVTZ//B3LYP/6-31G* (red)

# **S4. DP4 Calculations**

CP3 and DP4 are valuable methods to determine the candidate molecule with which the structure matches the experimental data the best, particularly in computational NMR structure elucidations and determination of relative stereochemistry of compounds. CP3 [[1](#_ENREF_1)] is used when two or more sets of experimental NMR data are available and DP4 [[2](#_ENREF_2)] is used when one set of experimental NMR data is available to many candidate structures.

In our study, molecule identification refers the process of matching of a set of sample NMR signals (^13^C and/or ^1^H chemical shifts) to the sets of NMR signals of candidates in a library or database. We assume to have one set of experimental NMR data (i.e. each set belongs to one molecule) to match to those of several candidate molecules. DP4 is more suitable to decide which candidate structure is the most likely to be correct than CP3.

We could have used MAE, CMAE, Pearson’s r or any other parameter to quantify the agreement between two sets. However, we believe that an indication of how confident a matching set is can be expressed better in terms of RMSE and probability. Formerly, we found RMSE to be a more appropriate measure of error than the MAE or others since each error does not contribute to RMSE in proportion to the absolute value of the error. Instead, the effects of squaring the error give more weight to errors further away from the mean, driving the error estimate towards the odd outlier. Latterly, the observation of weighted errors happens for DP4 in a similar way that standard cumulative normal distribution function yields more weight to larger errors than smaller ones, enabling to eliminate incorrect assignments in structure elucidations. Also, Smith et al. [[2](#_ENREF_2)] shows how DP4 is more successful at assigning stereochemistry than probabilities based on MAE and R. This is because DP4 is based on conditional probability and/or Bayes’ theorem--the key factor increasing the certainty of results. When we compared the results of two exact simulations, one with RMSE and the other with DP4, we found that DP4 gives slightly better rankings for pure samples while taking more computational time than RMSE. On the other hand, we believe DP4 is not convenient for ranking matches in impure/complex samples and computationally more intense than RMSE. Therefore, we use RMSE in this study.

In Case I, we have a pure sample where NMR signals (^13^C and/or ^1^H chemical shifts) belong to only one compound. We use the equation 1 for each matching of which candidates have equal number of ^13^C and/or ^1^H chemical shifts.

|  | $P\left( i\vert\delta_{1},\delta_{2},\ldots,\delta_{M} \right)=\frac{\prod_{k=1}^{N} (1-\Phi(\left\vert\left( \delta_{calc,k}^{i}-\delta_{exp,k}^{i} \right)-\mu\right\vert/\sigma))}{\sum_{j=1}^{M} [\prod_{k=1}^{N} (1-\Phi(\left\vert\left( \delta_{calc,k}^{i}-\delta_{exp,k}^{i} \right)-\mu\right\vert/\sigma))]}$ | (1) |
| --- | --- | --- |

where i is the candidate structure out of M possible candidates, $\Phi$(x) is the standard cumulative normal distribution function and k is the ^13^C or ^1^H nucleus (from 1 to N).

We performed a comparison study, DP4 versus RMSE, to decide whether DP4 is superior in predicting the best candidate correctly. We applied 100 different Gaussian noise ranging from 1.0 to 5.0 ppm and 0.2 to 4.0 ppm for ^13^C and/or ^1^H NMR chemical shifts of Set I, respectively. We repeated this procedure twice with the same dataset and conditions using DP4 and RMSE. DP4 could predict the molecule correctly for 93% of the runs and RMSE could do so for 92% of those while DP4 is 17% slower on average.

In Case II and Case III, we have an impure sample where NMR signals (^13^C and/or ^1^H chemical shifts) belong to many compounds. We use the equation 2, a slightly different version of equation 1, for each matching of which candidates have equal or a smaller number of ^13^C and/or ^1^H chemical shifts.

|  | $P\left( i\vert\mathrm{RMSD}_{1},\mathrm{RMSD}_{2},\ldots,\mathrm{RMSD}_{M} \right)=\frac{(1-\Phi(\left\vert\mathrm{RMSD}_{i}-\mu_{r} \right\vert/\sigma_{r}))\prod_{j\neq i} (1-\Phi(\left\vert\mathrm{RMSD}_{i}-\mu_{w} \right\vert/\sigma_{w}))}{\sum_{k=1}^{M} [(1-\Phi(\left\vert\mathrm{RMSD}_{i}-\mu_{r} \right\vert/\sigma_{r}))\prod_{j\neq i} (1-\Phi(\left\vert\mathrm{RMSD}_{i}-\mu_{w} \right\vert/\sigma_{w}))]}$ | (2) |
| --- | --- | --- |

where $\mu_{r}$ and $\mu_{w}$ , and $\sigma_{r}$and $\sigma_{w}$ stand for right and wrong assignment.

What is different herein is RMSE of whole nuclei is used instead of errors of individual nucleus. It is necessary due to the fact that a parameter is needed to normalize the products in the nominator since sizes of candidate structures are different. Any parameter could be used here such as MAE, R and etc. As we stated in the paper, we have preferred RMSE since it is better at giving a relatively high weight to undesired large errors than MAE. The use of standard cumulative normal distribution function yields less weight to smaller RMSEs and higher probability to them, but it does not affect the order of RMSEs (smallest to largest) or probabilities (largest to smallest), instead takes more computational time. Therefore, it is definitely better to use RMSE for Case II and Case III.

We could use DP4 for Case I for the sake of better identification, but we prefer RMSE instead since it provides better efficiency in computational time and effort, and to be consistent with the calculations for Case II.

Note that Smith et al recommends to empirically scale all the predicted NMR chemical shifts to remove any systematic errors in a DP4 analysis. Since a surrogate data is used in this study, the NMR data was not scaled. It is also assumed that calculated chemical shifts’ errors obey normal (Gaussian) distribution.

# **S5. Approach to determine the level of accuracy to identify molecules**

In the process of identification of compounds in a mixture, we followed the steps in the flowchart provided in the main manuscript (Figure 2). For clarifying the procedure, these steps are explained through an example molecule.

The molecule is oxaloacetamide with the molecular formula C_4_H_5_NO_4_ (IUPAC name is 4-amino-2,4-dioxobutanoic acid).

InChI is InChI=1S/C4H5NO4/c5-3(7)1-2(6)4(8)9/h1H2,(H2,5,7)(H,8,9).

InChIKey is ONGPAWNLFDCRJE-UHFFFAOYSA-N.

**Step I:** Generation of surrogate experimental NMR chemical shifts

**Step II:** Generation of computed NMR chemical shifts

|  |  | **Step I** | **Step II** | |
| --- | --- | --- | --- | --- |
| **No** | **Nuclei** | **Surrogate experimental chemical shifts (ppm)** | Gaussian distributed noise (ppm) is added | **Computed chemical shifts (ppm)** |
| 1 | C | 50.80 | **+**  σ = 1.0 ppm 🡪 | 51.70 |
| 2 | C | 195.19 |  | 195.77 |
| 3 | C | 162.29 |  | 162.14 |
| 4 | C | 163.14 |  | 163.72 |
| 10 | H | 3.86 | **+**  σ = 0.2 ppm 🡪 | 3.73 |
| 11 | H | 3.86 |  | 3.69 |
| 12 | H | 7.09 |  | 6.93 |
| 13 | H | 13.53 |  | 13.73 |
| 14 | H | 8.15 |  | 8.13 |

**Step III:** Matching the molecules of two sets: computed and surrogate experimental

The molecule taken from the computed data is searched in the surrogate experimental data. It is repeated for every single molecule in the database. ^13^C and ^1^H NMR chemical shifts are matched separately. The chemical shifts are sorted from smallest to largest and the closest values in each set are assigned to each other.

- Matching ^13^C NMR chemical shifts:

| **No** | **Nuclei** | **Surrogate experimental chemical shifts (ppm)** | **Matchings** | **Computed chemical shifts (ppm)** |
| --- | --- | --- | --- | --- |
| 1 | C | 50.80 | 🡪 | 51.70 |
| 3 | C | 162.29 | 🡪 | 162.14 |
| 4 | C | 163.14 | 🡪 | 163.72 |
| 2 | C | 195.19 | 🡪 | 195.77 |

- Matching ^1^H NMR chemical shifts

| **No** | **Nuclei** | **Surrogate experimental chemical shifts (ppm)** | **Matchings** | **Computed chemical shifts (ppm)** |
| --- | --- | --- | --- | --- |
| 10 | H | 3.86 | 🡪 | 3.69 |
| 11 | H | 3.86 | 🡪 | 3.73 |
| 12 | H | 7.09 | 🡪 | 6.93 |
| 14 | H | 8.15 | 🡪 | 8.13 |
| 13 | H | 13.53 | 🡪 | 13.73 |

**Step IV:** Evaluation of matching performance

For the molecule, the RMSE is calculated between the computed and surrogate experimental chemical shifts. It is repeated for every molecule in the dataset.

- RMSE for ^13^C NMR chemical shifts

| **No** | **Nuclei** | **Surrogate experimental chemical shifts (ppm)** | **Computed chemical shifts (ppm)** | **Error (ppm)** | **(Error)^2^ (ppm)** |
| --- | --- | --- | --- | --- | --- |
| 1 | C | 50.80 | 51.70 | 0.90 | 0.81 |
| 3 | C | 162.29 | 162.14 | -0.15 | 0.0225 |
| 4 | C | 163.14 | 163.72 | 0.58 | 0.3364 |
| 2 | C | 195.19 | 195.77 | 0.58 | 0.3364 |
|  | | | | **RMSE** | 0.376325 ~ 0.38 |

- RMSE for ^1^H NMR chemical shifts

| **No** | **Nuclei** | **Surrogate experimental chemical shifts (ppm)** | **Computed chemical shifts (ppm)** | **Error (ppm)** | **(Error)^2^ (ppm)** |
| --- | --- | --- | --- | --- | --- |
| 10 | H | 3.86 | 3.69 | -0.17 | 0.0289 |
| 11 | H | 3.86 | 3.73 | -0.13 | 0.0169 |
| 12 | H | 7.09 | 6.93 | -0.16 | 0.0256 |
| 14 | H | 8.15 | 8.13 | -0.02 | 0.0004 |
| 13 | H | 13.53 | 13.73 | 0.20 | 0.04 |
|  | | | | **RMSE** | 0.02795 ~ 0.03 |

After finding the RMSE values for ^13^C and ^1^H NMR chemical shifts, their geometric mean is found as:

RMSE = (0.38*0.03)^0.5^ = 0.01

**Step V:** Ranking scores

The RMSE scores of the molecules are sorted from the smallest to the largest. The top molecule (rank: 1) becomes the candidate compound.

# **S6. Current commonly used chemical databases**

- PubChem [[3](#_ENREF_3)]
- Royal Society of Chemistry ChemSpider [[4](#_ENREF_4)]
- ChEMBL by European Molecular Biology Laboratory [[5](#_ENREF_5), [6](#_ENREF_6)]
- Chemical Entities of Biological Interest (ChEBI) [[7](#_ENREF_7), [8](#_ENREF_8)]
- DrugBank [[9](#_ENREF_9), [10](#_ENREF_10)]
- Biological Magnetic Resonance Bank (BMRB) [[11](#_ENREF_11)]
- Human Metabolome Database (HMDB) [[12](#_ENREF_12)]
- GDB13 [[13](#_ENREF_13)]
- The Small Molecule Pathway Database (SMPDB) [[14](#_ENREF_14), [15](#_ENREF_15)]
- Distributed Structure-Searchable Toxicity (DSSTox) Database [[16](#_ENREF_16)]
- E. coli Metabolome Database (ECMDB) [[17](#_ENREF_17), [18](#_ENREF_18)]
- EcoCyc E. coli Database [[19](#_ENREF_19)]
- Food Component Database (FooDB) [[20](#_ENREF_20)]
- LIPID MAPS In-Silico Structure Database (LMISSD) [[21](#_ENREF_21)]
- MetaCyc Metabolic Pathway Database [[22](#_ENREF_22)], MolMall [[23](#_ENREF_23)]
- Super Natural II [[24](#_ENREF_24)]
- The Toxin and Toxin Target Database (T3DB) [[25](#_ENREF_25), [26](#_ENREF_26)]
- ToxCast [[27](#_ENREF_27)]
- The Universal Natural Products Database (UNPD) [[28](#_ENREF_28)]
- ZINC [[29](#_ENREF_29)]

**References**

1. Smith, S.G. and J.M. Goodman, *Assigning the stereochemistry of pairs of diastereoisomers using GIAO NMR shift calculation.* J Org Chem, 2009. **74**(12): p. 4597-607.

2. Smith, S.G. and J.M. Goodman, *Assigning stereochemistry to single diastereoisomers by GIAO NMR calculation: the DP4 probability.* J Am Chem Soc, 2010. **132**(37): p. 12946-59.

3. Peironcely, J.E., et al., *Automated pipeline for de novo metabolite identification using mass-spectrometry-based metabolomics.* Anal Chem, 2013. **85**(7): p. 3576-83.

4. Li, F., et al., *Stable isotope- and mass spectrometry-based metabolomics as tools in drug metabolism: a study expanding tempol pharmacology.* J Proteome Res, 2013. **12**(3): p. 1369-76.

5. Cripps, S.C., R.S. Orton, and J.E. Carroll, *Combined Theoretical and Experimental Studies of a Push-Pull Trapatt Circuit.* International Journal of Electronics, 1974. **37**(1): p. 1-21.

6. Gaulton, A., et al., *ChEMBL: a large-scale bioactivity database for drug discovery.* Nucleic Acids Res, 2012. **40**(Database issue): p. D1100-7.

7. Izgi, T., et al., *FT-IR and NMR investigation of 2-(1-cyclohexenyl)ethylamine: A combined experimental and theoretical study.* Spectrochimica Acta Part a-Molecular and Biomolecular Spectroscopy, 2007. **68**(1): p. 55-62.

8. de Matos, P., et al., *Chemical Entities of Biological Interest: an update.* Nucleic Acids Research, 2010. **38**: p. D249-D254.

9. Kwan, E.E. and R.Y. Liu, *Enhancing NMR Prediction for Organic Compounds Using Molecular Dynamics.* Journal of Chemical Theory and Computation, 2015. **11**(11): p. 5083-5089.

10. Knox, C., et al., *DrugBank 3.0: a comprehensive resource for 'Omics' research on drugs.* Nucleic Acids Research, 2011. **39**: p. D1035-D1041.

11. Ulrich, E.L., et al., *BioMagResBank.* Nucleic Acids Research, 2008. **36**: p. D402-D408.

12. Wishart, D.S., et al., *HMDB: a knowledgebase for the human metabolome.* Nucleic Acids Res, 2009. **37**(Database issue): p. D603-10.

13. Blum, L.C. and J.L. Reymond, *970 million druglike small molecules for virtual screening in the chemical universe database GDB-13.* J Am Chem Soc, 2009. **131**(25): p. 8732-3.

14. Jewison, T., et al., *SMPDB 2.0: big improvements to the Small Molecule Pathway Database.* Nucleic Acids Res, 2014. **42**(Database issue): p. D478-84.

15. Frolkis, A., et al., *SMPDB: The Small Molecule Pathway Database.* Nucleic Acids Res, 2010. **38**(Database issue): p. D480-7.

16. Richard, A.M. and C.R. Williams, *Distributed structure-searchable toxicity (DSSTox) public database network: a proposal.* Mutat Res, 2002. **499**(1): p. 27-52.

17. Guo, A.C., et al., *ECMDB: the E. coli Metabolome Database.* Nucleic Acids Res, 2013. **41**(Database issue): p. D625-30.

18. Sajed, T., et al., *ECMDB 2.0: A richer resource for understanding the biochemistry of E. coli.* Nucleic Acids Res, 2016. **44**(D1): p. D495-501.

19. Keseler, I.M., et al., *The EcoCyc database: reflecting new knowledge about Escherichia coli K-12.* Nucleic Acids Research, 2017. **45**(D1): p. D543-D550.

20. Scalbert, A., et al., *Databases on Food Phytochemicals and Their Health-Promoting Effects.* Journal of Agricultural and Food Chemistry, 2011. **59**(9): p. 4331-4348.

21. Fahy, E., et al., *Update of the LIPID MAPS comprehensive classification system for lipids.* Journal of Lipid Research, 2009. **50**: p. S9-S14.

22. Caspi, R., et al., *The MetaCyc database of metabolic pathways and enzymes.* Nucleic Acids Research, 2018. **46**(D1): p. D633-D639.

23. *MolMall*. [cited 2019 8/1]; Available from: <http://www.molmall.net/>.

24. Banerjee, P., et al., *Super Natural II-a database of natural products.* Nucleic Acids Research, 2015. **43**(D1): p. D935-D939.

25. Wishart, D., et al., *T3DB: the toxic exposome database.* Nucleic Acids Res, 2015. **43**(Database issue): p. D928-34.

26. Lim, E., et al., *T3DB: a comprehensively annotated database of common toxins and their targets.* Nucleic Acids Research, 2010. **38**: p. D781-D786.

27. Richard, A.M., et al., *ToxCast Chemical Landscape: Paving the Road to 21st Century Toxicology.* Chemical Research in Toxicology, 2016. **29**(8): p. 1225-1251.

28. Gu, J.Y., et al., *Use of Natural Products as Chemical Library for Drug Discovery and Network Pharmacology.* Plos One, 2013. **8**(4).

29. Sterling, T. and J.J. Irwin, *ZINC 15-Ligand Discovery for Everyone.* Journal of Chemical Information and Modeling, 2015. **55**(11): p. 2324-2337.
